# Comprehensive evolutionary analysis of growth-regulating factor gene family revealing the potential molecular basis under multiple hormonal stress in *Gramineae* crops

**DOI:** 10.3389/fpls.2023.1174955

**Published:** 2023-03-31

**Authors:** Wei Wang, Mingxing Cheng, Xiao Wei, Ruihua Wang, Fengfeng Fan, Zhikai Wang, Zhihong Tian, Shaoqing Li, Huanran Yuan

**Affiliations:** ^1^ State Key Laboratory of Hybrid Rice, Key Laboratory for Research and Utilization of Heterosis in Indica Rice of Ministry of Agriculture, Engineering Research Center for Plant Biotechnology and Germplasm Utilization of Ministry of Education, College of Life Sciences, Wuhan University, Wuhan, China; ^2^ Hubei Hongshan Laboratory, Wuhan, China; ^3^ College of Life Science, Yangtze University, Jingzhou, China

**Keywords:** GRFS, *Gramineae* crops, evolutionary analysis, transcriptional activators, hormone treatments

## Abstract

*Growth-regulating factors* (*GRFs*) are plant-specific transcription factors that contain two highly conserved QLQ and WRC domains, which control a range of biological functions, including leaf growth, floral organ development, and phytohormone signaling. However, knowledge of the evolutionary patterns and driving forces of *GRFs* in *Gramineae* crops is limited and poorly characterized. In this study, a total of 96 *GRFs* were identified from eight crops of *Brachypodium distachyon*, *Hordeum vulgare*, *Oryza sativa* L. ssp. *indica*, *Oryza rufipogon*, *Oryza sativa* L. ssp. *japonica*, *Setaria italic*, *Sorghum bicolor* and *Zea mays*. Based on their protein sequences, the *GRFs* were classified into three groups. Evolutionary analysis indicated that the whole-genome or segmental duplication plays an essential role in the *GRFs* expansion, and the *GRF*s were negatively selected during the evolution of *Gramineae* crops. The GRFs protein function as transcriptional activators with distinctive structural motifs in different groups. In addition, the expression of *GRFs* was induced under multiple hormonal stress, including IAA, BR, GA3, 6BA, ABA, and MeJ treatments. Specifically, *OjGRF11* was significantly induced by IAA at 6 h after phytohormone treatment. Transgenic experiments showed that roots overexpressing *OjGRF11* were more sensitive to IAA and affect root elongation. This study will broaden our insights into the origin and evolution of the GRF family in *Gramineae* crops and will facilitate further research on *GRF* function.

## Introduction

*Growth-regulating factors* (*GRFs*) are plant-specific transcription factors that are widely distributed in the plant kingdom. *OsGRF1* was the first identified member of the GRF family in plants and is known to play a crucial role in early stem elongation (Knaap and Kende, 2000). Since its discovery, GRF proteins have been gradually identified in other plant species, such as *Arabidopsis thalian*a, *Brassica napus*, *Glycine ma*x, and *Solanum tuberosum* (Greco et al., 2012; Kim et al., 2012; Omidbakhshfard et al., 2015; Zhang et al., 2018). GRF proteins typically range in size from 20 to 60 kD and commonly contain two highly conserved domains: QLQ and WRC (Vercruysse et al., 2020). However, the composition and number of amino acid residues in the C-terminal region vary greatly (Kim and Tsukaya, 2015). The QLQ domain, consisting of 36 amino acid residues with highly conserved Gln-Leu-Gln (QX3LX2Q) residues, is a crucial motif for protein-protein interactions (Knaap and Kende, 2000). In contrast, the WRC domain is typically associated with DNA binding and contains a putative nuclear localization signal (Omidbakhshfard et al., 2015). This domain consists of 44 amino acid residues and is characterized by a Trp-Ar-Cys motif and a conserved distance between three cysteine residues and one histidine residue (CX9CX10CX2H; C3H motif) (Kim and Tsukaya, 2015).

Transcription factors are essential regulators of gene expression, and rice possesses approximately 2147 transcription factors (Jin et al., 2017). Among them, *GRFs* are major transcription factors in plants that play a crucial role in various developmental processes, including regulating the growth of plant roots, leaves, and floral organ *GRF9*, a negative regulator of leaf growth, reduces leaf development when overexpressed, whereas *grf9* mutants produce larger leaf primordia, rosette leaves, and petals. *GRF9* activates the expression of LEUCINE-ZIPPER (*bZIP*) type transcription factor *OBP3-RESPONSIVE GENE3* (*ORG3*) to regulate leaf growth. In flowers, *APETALA1* (*AP1*) and *SEPALLATA3* (*SEP3*) are the main controlling factors. (Kaufmann et al., 2009). They interact specifically with members of the GRF family to regulate floral organ development (Pajoro et al., 2014). *AtGRF2* and *AtGRF5* are highly expressed in floral organ meristem and primary primordium, while *AtGRF8* is expressed in floral organ differentiation (Pajoro et al., 2014). In maize, overexpression of *ZmGRF10* reduced leaf size and plant height by significantly decreasing the expression of *PIF4* (*phytochrome interaction factor 4*), *TATA-binding protein-related factor 5* (*TAF5*) and *REVISRADIX* (*BRX*) genes (Wu et al., 2014).

In rice, the *GRFs* are regulated by *OsmiR396*, including *OsGRF4*, *OsGRF6*, and *OsGRF10*, which play important roles in regulating grain size, grain yield, and inflorescence development (Liu et al., 2014; Che et al., 2015; Duan et al., 2015; Gao et al., 2015; Hu et al., 2015; Li et al., 2016; Tang et al., 2018). Down-regulation of target *OsGRFs* by 35S: osa-miR396 leads to a dehiscent outer shell of the floret, elongated and sterile lemma, abnormal number of anthers, and stigma in progeny plants (Liu et al., 2014). In OEmiR396b plants, *miR396* inhibits the expression of *OsGRF6* by cleaving its transcripts, leading to shortened branches, clusters, and decreased yield (Gao et al., 2015). *OsGRF4* is regulated by *miR396c*, and the mutation of *OsGRF4* suppresses the regulation of the *OsmiR396* target. Additionally, the direct interaction between *OsGRF4* and *OsGIF1* results in larger grains and improved yield of rice (Li et al., 2016).

One of the most important regulators of plant growth and development, *GRFs* have been reported in several cereal crops, including rice, wheat, and corn (Zhang et al., 2008; Greco et al., 2012; Kim et al., 2012; Fİlİz et al., 2014; Omidbakhshfard et al., 2015). However, a systematic understanding of the evolution of the GRF family in *Gramineae* crops is still lacking. In this study, the *GRFs* in the *Gramineae* crops of *Brachypodium distachyon*, *Hordeum vulgare*, *Oryza sativa* L. ssp. *indica*, *Oryza rufipogon*, *Oryza sativa* L. ssp. *japonica*, *Setaria italic*, *Sorghum bicolor* and *Zea mays* were identified, and the exon-intron structure, chromosomal distribution and the evolution of *GRFs* were systematically characterized. This study will help to deepen our understanding of the function and evolutionary relationship of *GRF*s in the *Gramineae* crops.

## Materials and methods

### Identification of *GRF* family members from *Gramineae* crops

A total of eight genomes of *Gramineae* crops, including *Brachypodium distachyon*, *Hordeum vulgare*, *Oryza sativa* L. ssp. *indica*, *Oryza rufipogon*, *Oryza sativa* L. ssp. *japonic*a, *Setaria italic*, *Sorghum bicolor* and *Zea mays*, were downloaded from Ensembl Plants release-41 (http://plants.ensembl.org/index.html) and Phytozome v.12 (https://phytozome-next.jgi.doe.gov/) (Goodstein et al., 2012; Bolser et al., 2016). To identify *GRFs* in the eight *Gramineae* crops, PF08879 and PF08880 were downloaded from InterPro (www.ebi.ac.uk/interpro). Then all the *GRF* candidate genes were separately identified by HMMER 3.2.1 and BLASTP (Camacho et al., 2009; Finn et al., 2011). Finally, the sequences were further verified by using InterPro (www.ebi.ac.uk/interpro) and SMART (http://smart.embl-heidelberg.de) (Malik et al., 2020; Cheng et al., 2021).

### Gene structure, motif and phylogenetic analysis

To identify the exon/intron of the conserved GRFs protein, we extracted eight genomes annotation GFF files from Ensembl Plants website (Bolser et al., 2016). The software MEME Suite v.5.3.3 was used to identify conserved motifs with a maximum number of 20, the phylogenetic tree was constructed by MEGA and then visualized and managed by EvolView (He et al., 2016), motif arrangement and the exon/intron structures were shown by TBtools (Chen et al., 2020a).

The full-length amino acid sequence of the GRFs from the eight plant genomes were aligned with the ClustalW program (version 2.0) and constructed the neighbor-joining (NJ) tree with 1000 bootstrap replicates using the Poisson substitution model in MEGA 7.0 (Edgar and Sjolander, 2004; Kumar et al., 2016).

### Chromosomal locations, gene duplication analysis and Orthogroup analysis

Synteny relationship between duplicated gene pairs from eight *Gramineae* crops genomes was analyzed by using MCScanX software and diagrammatical results were visualized by using simple Circos-0.69 software (http://circos.ca/) (Krzywinski et al., 2009; Wang et al., 2012).

The orthogroup was identified using OrthoFinder v.2.5.4 with a cut-off e-value of 1×10^−3^ (Emms and Kelly, 2019). Then, STAG and STRID algorithms were used to rebuild the phylogenetic tree of the selected species based on the detected orthogroup (Emms and Kelly, 2019).

### Collinearity analysis and calculation of selection pressure of *GRFs*

To investigate the collinearity and analyze the syntenic relationship of *GRFs* in eight *Gramineae* crops, the whole genome sequences and corresponding genome annotation files *GRFs* were analyzed by the MCScanX tool (Wang et al., 2012). For the calculation of the selection pressure of *GRFs* among eight species, synonymous (Ks) and non-synonymous (Ka) substitution rates and their ratios were estimated using the Ka/Ks calculator in TBtools (Cheng et al., 2021). And the divergence time (T) was estimated by T = Ks/(2ⅹ9.1ⅹ10^−9^)ⅹ10^−6^ million years ago (MYa) (Cheng et al., 2021).

### Analysis of cis-acting elements and miRNAs targeting *GRF* family members

According to the DNA sequence 2000 bp upstream of the *GRFs* in *japonica* rice, the cis-acting elements were predicted from the PlantCARE website (http://bioinformatics.psb.ugent.be/webtools/plantcare/html) (Rombauts et al., 1999) and categorized based on their annotated functions. Furthermore, the other miRNAs except for *miR396* potentially target to *GRFs* were predicted using the website psRNATarget (https://www.zhaolab.org/psRNATarget/home).

### Hormone treatments and RT-qPCR analysis

The rice cultivar Zhong-Hua-11 (ZH11, *Oryza sativa* L. ssp. *japonica*) was used to analysis the expression of *GRF*s. The rice plants were soaked in 100 µM hormone (IAA, BR, GA3, 6BA, ABA, and MeJ) at the three-leaf-one-heart stage for various time points (0 h, 6 h, 9 h, 12 h, 24 h). All the seedlings were cultured at 28 °C for 12 days with 14-h light/10-h dark and 60% relative humidity (Cheng et al., 2021).

All RNAs of samples were extracted using TRIzol Reagent according to the manufacturer’s protocol (Invitrogen, Carlsbad, CA, USA), And the first-strand cDNA was synthesized from RNA treated with a 2 µg DNA enzyme using HiScript III First Strand cDNA Synthesis Kit (+gDNA Wiper) (Vazyme, Nanjing, China). The RT-qPCR analysis of *GRF*s in rice was performed by using the HieffqPCR SYBR Green Master Mix (No Rox) (Yeasen, Shanghai, China), and the relative expression level of the 12 *GRF*s was analyzed with the 2^−ΔΔCT^ method. *GRF*s specific primers used for RT-qPCR analysis were listed in **Supplementary Table 8**.

### Subcellular localization of GRFs protein

The open reading frames (ORFs) of five *GRFs* in ZH11 (*Oryza sativa* L. ssp. *japonica*) were amplified from their cDNA and inserted into the modified HBT-eGFP vector to produce 35S: GRF: GFP fusion vector. The structure is driven by the CaMV 35S promoter. The recombinant plasmids were transformed into DH5α chemically competent cells (Weidi Biotechnology, Shanghai). The recombinant competent cells were transinfected into rice protoplasts. The empty HBT vector was used as a control, and all the protoplasts were cultured at 28 °C overnight. Then the protoplasts were observed for GFP signals by using the FV1000 confocal system. The *GRF*s specific primers for subcellular localization were listed in **Supplementary Table 9**.

### Analysis of the transcriptional activity of *GRFs*

To investigate the transcriptional activity of *GRFs*, the full-length coding sequences of *GRF*s were amplificated into pGBKT7, pGADT7, and GAL4DB vectors. The recombinant plasmids pGBKT7 and pGADT7 were transformed into yeast strain AH109 chemically competent cells (Weidi Biotechnology, Shanghai). The chemically competent cells were cultured on the corresponding defective medium for 3-5 days for observation. Then GRF-GAL4DB fusion proteins were transiently expressed in rice protoplasts to measure transcriptional activity by using the dual-luciferase reporter assay system (Promega, Madison, WI, USA) (Ren et al., 2016). The empty GAL4DB vector was used as a control. The specific primers used are listed in **Supplementarys Table 10**, **11**.

## Results

### Identification and characterization of *GRFs* in the *Gramineae* crops

The coding genes for *GRF* transcription factors were identified in *Brachypodium distachyon*, *Hordeum vulgare*, *Oryza sativa* L. ssp*. indica*, *Oryza rufipogon*, *Oryza sativa* L. ssp*. japonica*, *Setaria italic*, *Sorghum bicolor* and *Zea mays* by searching for the WRC domain (Pfam accession no. PF08879, InterPro accession no. IPR014977) and QLQ domain (Pfam accession no. PF008880, InterPro accession no. IPR014978) using Pfam (Mistry et al., 2021). A total of 96 *GRFs* were detected, with 12, 13, 13, 12, 12, 8, 10, and 16 *GRFs* identified in *Brachypodium distachyon*, *Hordeum vulgare*, *Oryza sativa* L. ssp*. indica*, *Oryza rufipogon*, *Oryza sativa* L. ssp*. japonica*, *Setaria italic*, *Sorghum bicolor* and *Zea mays* respectively (**Supplementary Table 1**). The number of *GRFs* is relatively stable among these eight *Gramineae* crops.

**Figure 1:**
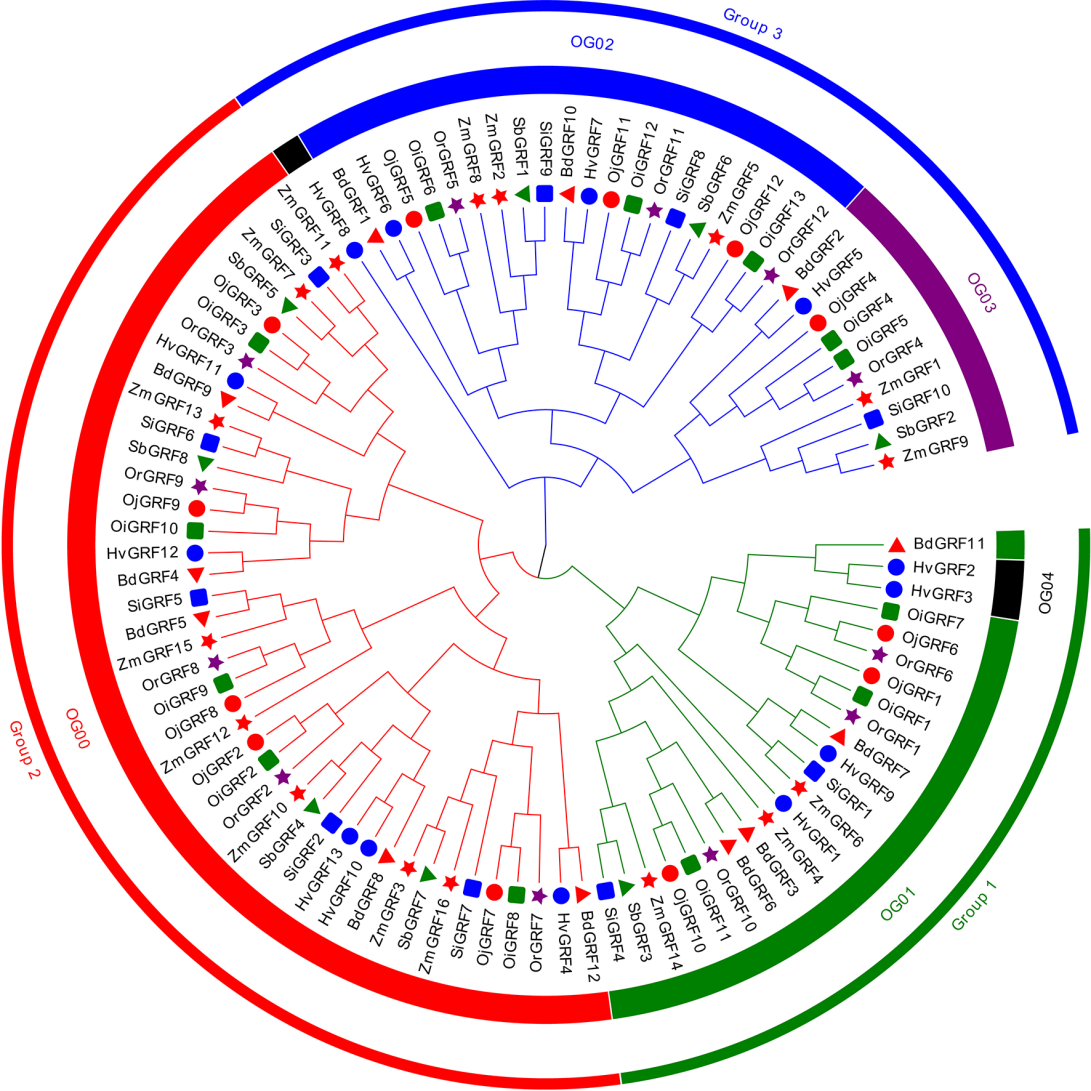
The neighbor-joining (NJ) phylogeny tree of the *GRFs* based on protein sequences from eight *Gramineae* crops. The colors of the outside circles represent different subfamilies, including Group1, Group2, and Group3. The colors of the inside circles represent different orthogroups (OGs). The different colors and shapes indicate the different species, the red triangle means *Brachypodium distachyon*, blue filled circles denote *Hordeum vulgare*, green squares represent *Oryza sativa* L. ssp. *indica*, red filled circles show *Oryza sativa* L. ssp. *japonica*, brown pentagon stand for *Oryza rufipogon*, blue squares mean *Sorghum bicolor*, the green triangle represents *Sorghum bicolor*, red pentagon denotes *Zea mays*.

To understand the phylogenetic relationship of the *GRF*s among *Gramineae* crops, the phylogenetic tree was constructed and classified the identified GRF protein sequences into three subfamilies: Group 1, Group 2, and Group 3 (**Figure 1**). All subgroups showed a clear expansion of gene numbers, with Group2 having the highest number of gene members, followed by Group1 and Group3 (**Figure 1** and **Supplementary Table 2**). In *Setaria italic*, *Sorghum bicolor* and *Zea mays* Group1 had fewer members (**Figure 1**, **Supplementary Table 2**). To better understand the evolutionary pattern of *GRF*s in *Gramineae* crops, we performed orthogroup (OG) clustering using OrthoFinder software 9, which divided the GRFs into five orthogroups: OG00, OG01, OG02, OG03, and OG04 (**Figure 1**). The number of genes varied greatly among the orthogroups, ranging from 3 to 42 (**Figure 1**, **Supplementarys Table 3**, **4**). Notably, the OG04 contained only three *GRF*s from *Hordeum vulgare*, indicating unequal loss and expansion of *GRFs* among orthogroups during the evolution of *Gramineae* crops.

### Expansion and evolutionary pattern of the *GRFs*

**Figure 2:**
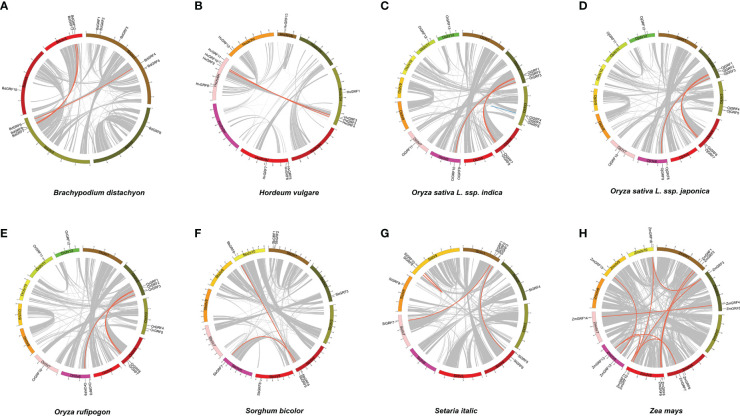
The chromosome location and duplication events of *GRFs* in eight *Gramineae* crops. The position of each *GRF* gene was marked using Circos software, and whole-genome duplication (WGD) or fragment duplication/tandem duplication gene pairs were connected by red/blue lines. **(A)** *Brachypodium distachyon*. **(B)** *Hordeum vulgare*. **(C)** *Oryza sativa* L. ssp. *indica*. **(D)** *Oryza rufipogon*. **(E)** *Oryza sativa* L. ssp. *japonica*. **(F)** *Setaria italic*. **(G)** *Sorghum bicolor*. **(H)** *Zea mays*.

To gain insight into the expansion mechanism of *GRFs* among the eight *Gramineae* crops, we further analyzed the chromosomal distribution and duplication modes of the *GRF*s. The *GRFs* were found to be mainly distributed on 3-4 chromosomes in each species (**Figure 2**), with chromosome 2 having the largest proportion, while chromosome 8 had only one *GRF* (**Supplementary Table 1**). In *Brachypodium distachyon*, nearly half of the *GRFs* were located on chromosome 1 (**Figure 2**), whereas in *Oryza sativa* L. ssp. *japonica*, the *GRF*s were almost evenly distributed on chromosomes 2, 3, 4, 6, 7, 11, and 12 (**Figure 2** and **Supplementary Table 1**). Additionally, a total of 33 duplicated gene pairs were identified in the eight species (**Table 1**), which were generated from either segmental or tandem duplication. However, tandem duplications were found only in *Hordeum vulgare* and *Oryza sativa* L. ssp. *indica* (**Table 1**). Importantly, the *GRF* number and the duplication mode were the same between *Oryza sativa* L. ssp*. Japonica* and *Oryza rufipogon*, indicating a close evolutionary relationship and supporting the notion that *Oryza rufipogon* is the direct ancestor of *Oryza sativa* (Londo et al., 2006).

**Table 1:** Ka and Ks values for the duplicated gene pairs in *Gramineae* crops.

Seq1	Seq2	Ks	Ka	Ka/Ks Ratio	Date (Mya)	Duplication type
BdGRF4	BdGRF9	0.3194	0.4411	0.7243	24.23	WGD or segmental duplications
BdGRF7	BdGRF11	0.2758	0.3408	0.8093	18.73	WGD or segmental duplications
BdGRF8	BdGRF12	0.2382	0.5435	0.4383	29.86	WGD or segmental duplications
HvGRF2	HvGRF3	0.0712	0.0823	0.8656	4.52	tandem duplication
HvGRF2	HvGRF9	0.3930	0.6394	0.6147	35.13	WGD or segmental duplications
HvGRF4	HvGRF10	0.2695	0.5143	0.5241	28.26	WGD or segmental duplications
OiGRF1	OiGRF7	0.1742	0.4567	0.3814	25.09	WGD or segmental duplications
OiGRF2	OiGRF8	0.1394	0.5711	0.2442	31.38	WGD or segmental duplications
OiGRF3	OiGRF10	0.2788	0.4027	0.6923	22.12	WGD or segmental duplications
OiGRF4	OiGRF5	0.0033	0.0041	0.7983	0.23	tandem duplication
OrGRF1	OrGRF6	0.1565	0.4463	0.3506	24.52	WGD or segmental duplications
OrGRF2	OrGRF7	0.1361	0.5820	0.2338	31.98	WGD or segmental duplications
OrGRF3	OrGRF9	0.2966	0.3917	0.7572	21.52	WGD or segmental duplications
OjGRF1	OjGRF6	0.1718	0.4726	0.3635	25.97	WGD or segmental duplications
OjGRF2	OjGRF7	0.1815	0.6595	0.2752	36.24	WGD or segmental duplications
OjGRF3	OjGRF9	0.2782	0.3564	0.7806	19.58	WGD or segmental duplications
SiGRF2	SiGRF7	0.1981	0.5327	0.3719	29.27	WGD or segmental duplications
SiGRF3	SiGRF6	0.3486	0.4673	0.7460	25.68	WGD or segmental duplications
SiGRF9	SiGRF10	0.5874	2.2248	0.2640	122.24	WGD or segmental duplications
SbGRF4	SbGRF7	0.2734	0.6779	0.4033	37.25	WGD or segmental duplications
SbGRF5	SbGRF8	0.3371	0.6886	0.4896	37.83	WGD or segmental duplications
ZmGRF1	ZmGRF9	0.0818	0.3999	0.2046	21.97	WGD or segmental duplications
ZmGRF2	ZmGRF8	0.0887	0.2288	0.3876	12.57	WGD or segmental duplications
ZmGRF3	ZmGRF10	0.2872	0.5516	0.5206	30.31	WGD or segmental duplications
ZmGRF3	ZmGRF16	0.0662	0.1995	0.3319	10.96	WGD or segmental duplications
ZmGRF4	ZmGRF14	0.0534	0.2781	0.1921	15.28	WGD or segmental duplications
ZmGRF7	ZmGRF13	0.3475	0.6997	0.4967	38.44	WGD or segmental duplications
ZmGRF7	ZmGRF11	0.0856	0.1639	0.5221	9.01	WGD or segmental duplications
ZmGRF10	ZmGRF16	0.2745	0.5467	0.5022	30.04	WGD or segmental duplications
ZmGRF11	ZmGRF13	0.3780	0.5578	0.6776	30.65	WGD or segmental duplications
ZmGRF12	ZmGRF15	0.0585	0.2329	0.2514	12.80	WGD or segmental duplications

Synonymous (Ks) and nonsynonymous (Ka) substitution rates of duplicate gene pairs (Ka/Ks ratios).

The investigation of divergence revealed a wide range of divergent times for the duplicated gene pairs, ranging from 0.23 to 122.24 Mya (**Table 1**), indicating significant variation in the origin of *GRFs* among *Gramineae* crops. For instance, the divergence time ranged from 25.68 to 122.24 Mya in *Setaria italic*, and 0.23 to 31.38 Mya in *Oryza sativa* L. ssp*. indica*. Ka and Ks analysis demonstrated that the Ka/Ks values were all less than 1 in the *Gramineae* crops (**Table 1**), suggesting that *GRFs* have been subjected to strong negative selection during the evolution of *Gramineae* crops.

### Syntenic relationship of *GRF*s among the *Gramineae* crops

**Figure 3:**
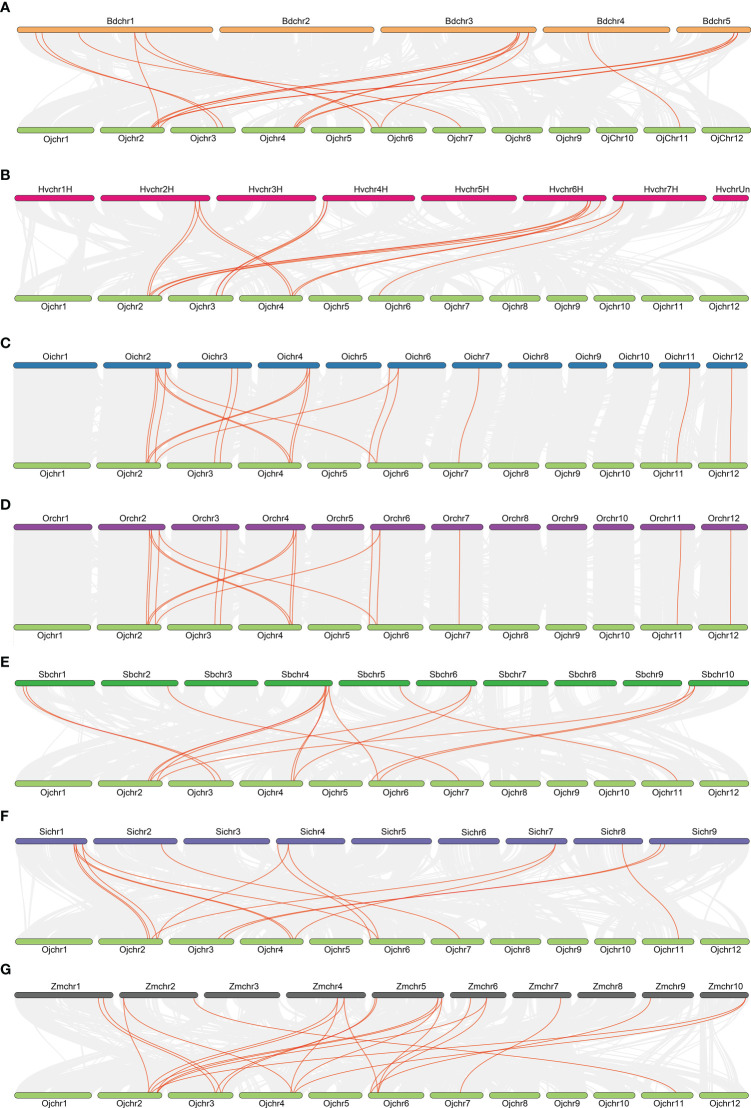
Collinearity relationships of GRFs in eight species or subspecies. Brachypodium distachyon **(A)**, Hordeum vulgare **(B)**, Oryza sativa L. ssp. indica, **(C)**, Oryza sativa L. ssp. japonica **(D)**, Sorghum bicolor **(E)**, Setaria italic **(F)**, Zea mays **(G)**.

To further elucidate the evolutionary relationship of the *GRF* family within the *Gramineae* crops, we analyzed the synteny of *GRF*s using the MCScanX toolkit. Reference with the genome of *japonica* rice Nipponbare, there were 17, 14, 18, 18, 15, 15 and 22 collinear gene pairs being detected in the genomes of *Brachypodium distachyon*, *Hordeum vulgare*, *Oryza sativa* L. ssp. *indica*, *Oryza rufipogon*, *Setaria italic*, *Sorghum bicolor*, and *Zea mays* (**Figure 3**). The collinearity of *GRF*s between the *Oryza sativa* L. ssp. *japonica* and *Oryza sativa* L. ssp. *indica*, *Oryza rufipogon* and *Zea mays* were closer than in the other species (**Figure 3**), consistent with their closer evolutionary ship. These results suggest the syntenic relationship of the *GRF*s is highly conserved among *Gramineae* crops.

### Gene structure and functional motifs of *GRFs*

**Figure 4:**
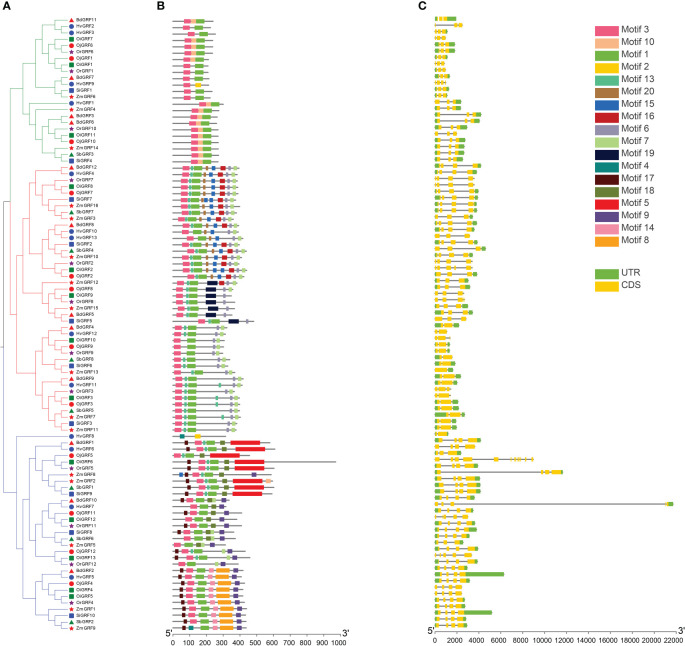
Phylogenetic relationships, gene structure, and the protein conserved motif of *GRFs* in eight *Gramineae* crops. **(A)** According to the NJ method in MEGA software, a phylogenetic tree of the *GRF* family from the eight *Gramineae* crops. **(B)** Distribution of the conserved motifs of the *GRFs* elucidated by TBtool Software. The different colored and numbered boxes represented different conserved motifs. **(C)** Exon-intron structures of *GRF* family. The green boxes represent exon and the gray line represents intron.

To elucidate the functional relationships of among members of the GRF family during evolution, the conserved protein motifs and structures of the 96 *GRFs* were analyzed. Results showed that the exon number of *GRF*s in *Gramineae* crops ranged from 2 to 5, except *OiGRF6* had 12 exons (**Figure 4C**). Moreover, more than 70% of *GRF*s consisted of three or four exons in each species (**Figures 4A, C**).

**Figure 5:**
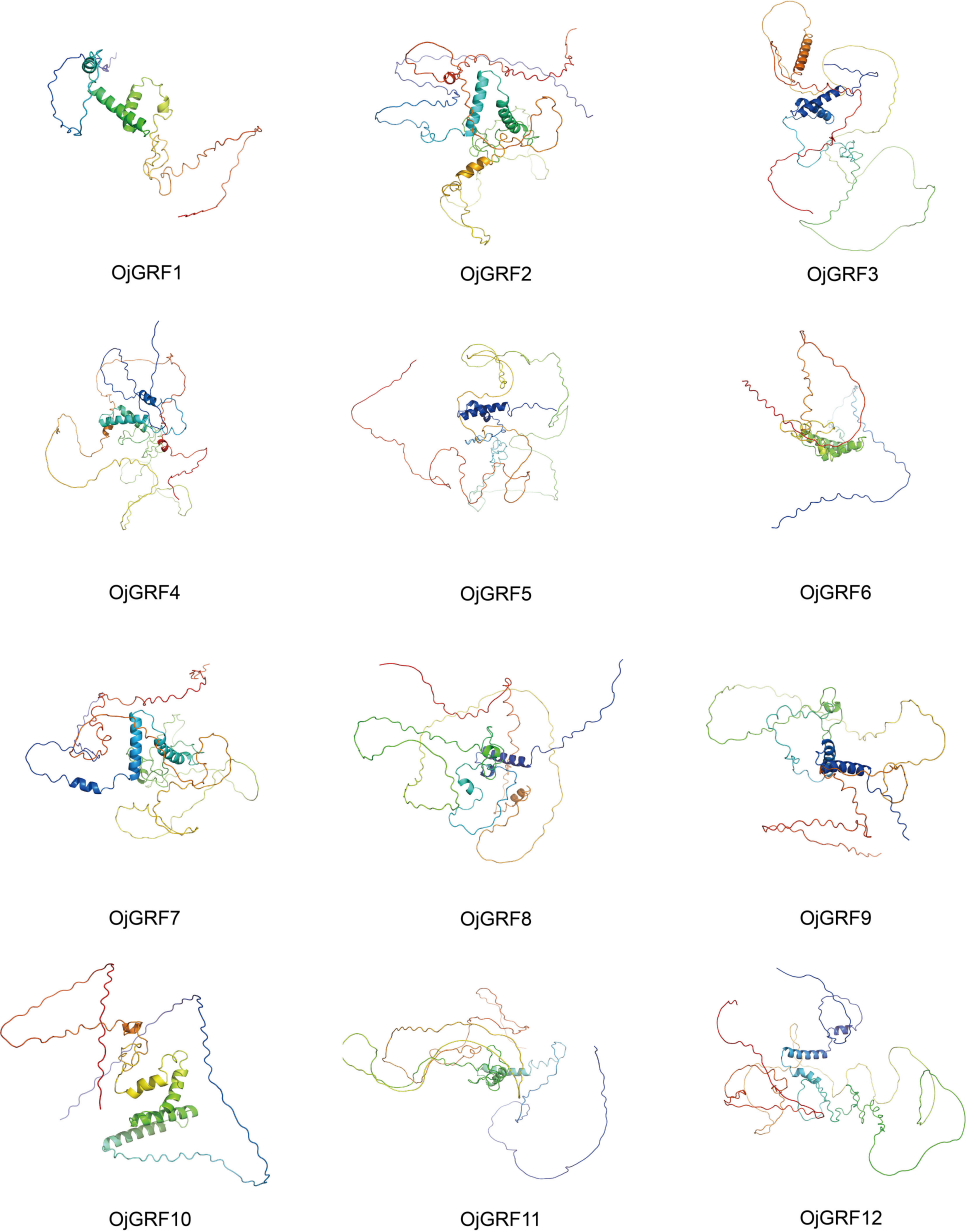
Prediction of the three-dimensional structure of GRFs protein in rice. The three-dimensional structure of 12 GRFs protein in rice was predicted by using the Alphafold2, and the results were output by PyMol software.

The secondary structure analysis showed the GRFs protein were mainly composed of Alpha helix, Extended strand, Beta turn, and Random coil (**Supplementary Table 5**). Specifically, Random coil accounted for 51.67~70.79%, followed by Alpha helix (13.76~33.46%), Extended strand (4.98~14.83%), and Beta turn (1.97~7.2%) (**Supplementary Table 5**). The three-dimensional structure analysis of the GRFs protein in rice with Alphafold2 (Jumper et al., 2021) showed that the GRFs protein were highly similar, with a simple arrangement lacking complex helical folded structures (**Figure 5**).

Functional motif analysis revealed that *GRFs* contain a total of 20 conserved motifs (**Figure 4B**). Most *GRFs* contain motif 1 and motif 3, and those with similar protein motif patterns were clustered into the same clade (**Figures 4A, B**). This is consistent with the phylogenetic analysis in **Figure 1**, indicating the sequence and functional conservation of *GRFs* within the same clade of *Gramineae* crops.

### Subcellular localization and transcriptional activity of *GRFs* in rice

**Figure 6:**
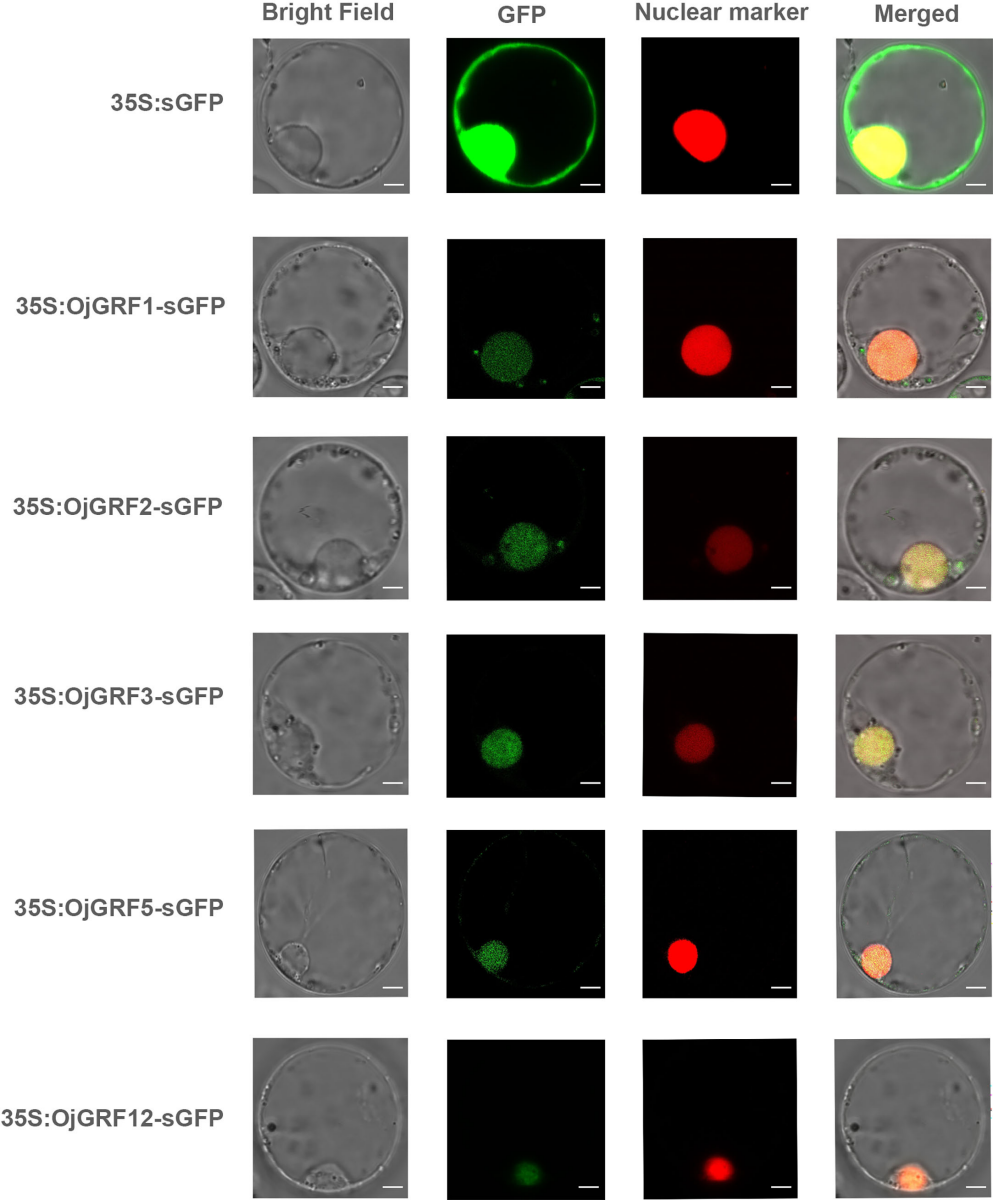
The subcellular localization of GRFs protein in rice protoplasts. The GRFs were fused to the GFP signal protein and the RFP signal represents the nucleus. The empty vector, HBT-sGFP, was used as a control. Scale bars correspond to 5 mm.

Transcription factors typically contain DNA binding domains (BD), transcriptional activation domains (AD), and nuclear location signal regions, which often form dimers to perform functions in the nucleus (Miura et al., 2010). As *GRFs* have been identified as important transcription factors in plants, it is of interest to determine whether all GRF proteins are localized in the nucleus. We predicted the 12 *GRFs* in *japonica* rice and found that all of them contained nuclear localization signals (**Supplementary Table 6**). Then, the *GRFs* were transiently expressed in the rice protoplast under driven by the CaMV 35s promoters, and found that the OjGRF1, OjGRF2, OjGRF3, OjGRF5 and OjGRF12 from *japonica* rice had green fluorescence signals in the nucleus (**Figure 6**), indicating that these *GRFs* are located in the nucleus.

**Figure 7:**
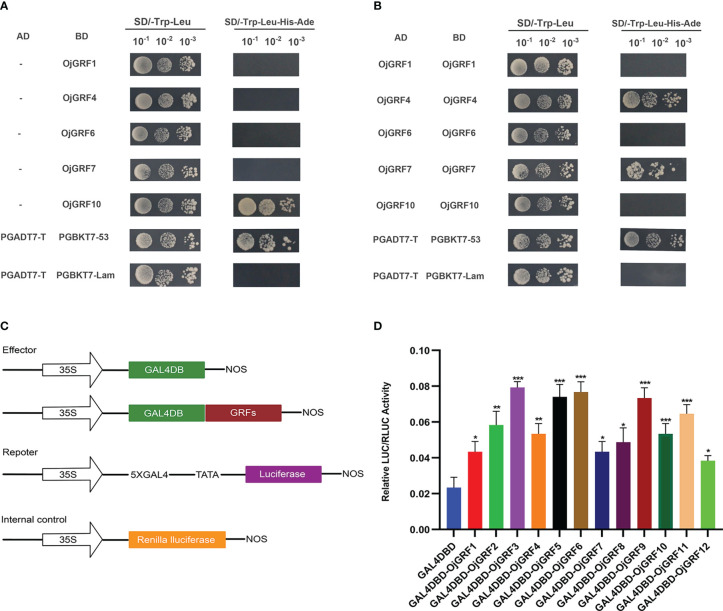
Transcriptional activity assay of *GRFs*. **(A)** Analysis of GRFs protein transcriptional self-activating activity in yeast; **(B)** Analysis of GRFs protein dimers in yeast; **(C)** The vector constructs are used in the dual-luciferase assay; **(D)** Dual-luciferase assay in rice protoplasts. The error bars show the standard deviations of the three independent biological replicates. Student’s t-test was used in this experiment; *; *p < 0.05, **p < 0.01, ***p < 0.001.

To investigate the transcriptional activity of GRFs, their coding regions were cloned into the pGBKT7 and pGADT7 vectors and tested by transforming yeast. Expression assay revealed that, except for OjGRF1, OjGRF4, OjGRF6 and OjGRF7 (**Figure 7A**), the other eight *GRFs* from *japonica* rice exhibited transcriptional self-activating activity (**Figure 6A** and **Supplementary Figure 1**). Moreover, nine GRFs protein from *japonica* rice were able to form dimers, except for OjGRF1, OjGRF6, and OjGRF10 (**Figure 7B** and **Supplementary Figure 2**). Transient expression assays with a dual-luciferase reporter in rice protoplasts showed that all the *GRFs* had transcriptional activity (**Figures 7C, D**). These results demonstrated that *GRFs* function as transcriptional activators.

### Identification of cis-acting elements of *GRF*s in *japonica* rice

**Figure 8:**
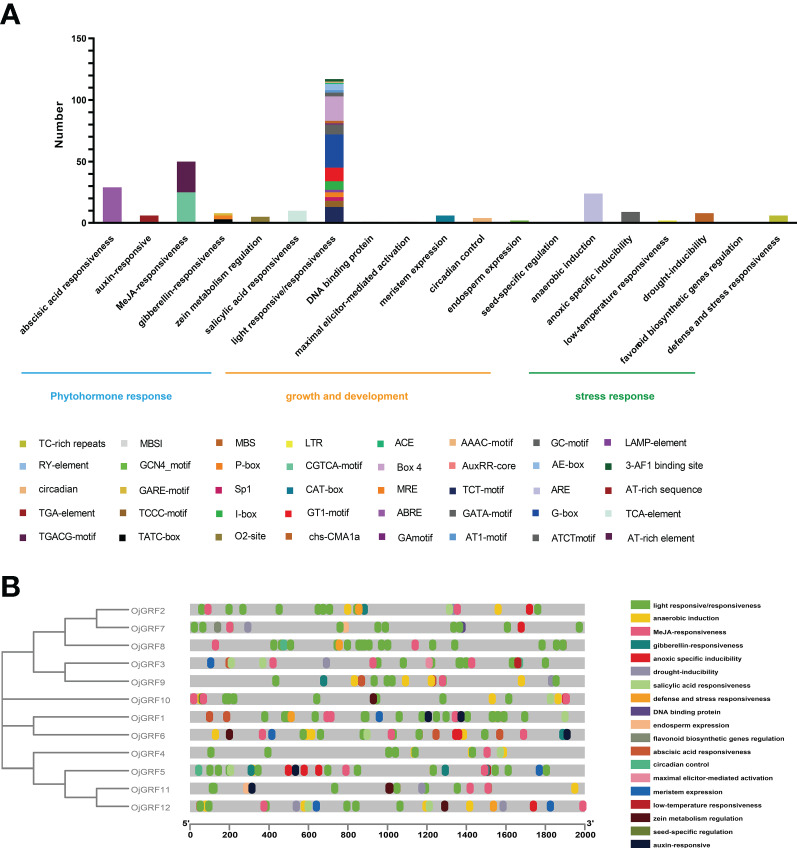
Identification of cis-acting elements in all *GRFs* of *japonica* rice. **(A)** Distribution of cis-acting elements in a different category. **(B)** Cis-acting elements of all *GRFs* in the phylogenetic tree. The differently colored boxes mean different promoter elements in each *GRF* gene.

Cis-acting elements in the promoters usually play an important role in responding to diverse environments and regulating gene-specific expression (Wang et al., 2018). Thus, the potential cis-acting elements in the promoter regions 2,000 bp upstream from the RNA start site of *GRF*s were identified using the PlantCARE database (Lescot et al., 2002). A total of 41 types of cis-acting elements were detected in the *GRF* promoters of the eight *Gramineae* crops, which were mainly classified into three categories: growth and development, phytohormone response, and stress response (**Figure 8A**).

The growth and development category contained 18 types of cis-acting elements including several elements involved in light responsiveness (3-AF1 binding site, AAAC-motif, ACE, AE-box, AT1-motif, ATCT-motif, Box 4, chs-CMA1a, GA-motif, GATA-motif, G-box, GT1-motif, I-box, LAMP-element, MRE, Sp1, TCCC-motif, TCT-motif). Among them, the G-box (23.1%) and Box 4 (17.1%) were the most abundant elements in this category, indicating that light responsiveness elements widespread distribution throughout the promoter region (**Figure 8A**). In the phytohormone response category, almost half of the predicted cis-acting elements were related to the MeJA-responsiveness, including CGTCA-motif and TGACG-motif. In addition, part of the elements related to abscisic acid responsiveness (ABRE), auxin-responsive (AuxRR-core and TGA-element), gibberellin-responsiveness (GARE-motif, P-box, and TATC-box), salicylic acid responsiveness (TCA-element), and zein metabolism were also observed (**Figure 8A**), implying that the *GRF*s play multiple roles in hormone response. Further analysis revealed that the cis-acting elements were unevenly distributed among the *GRF*s, and some cis-acting elements, such as GCN4_motif, MBSI, AT-rich sequence, and RY-element, were concentrated in a few *GRF*s (**Figure 8B**), reflecting the functional differentiation of the *GRFs*.

In plants, miRNAs play a crucial role in regulating various biological processes by binding to their target genes. The 12 *GRF*s in rice were predicted as the potential targets of 91 miRNAs. Among these, the *miR167*, *miR1861*, *miR395*, *miR399* and *miR395*, except *miR396*, were all predicted targeting to *GRF*s (**Supplementary Table 7**), reflecting the regulatory complexity of *GRF*s at RNA level.

### Expression profiles of the *GRF*s in rice under various hormonal treatments

**Figure 9:**
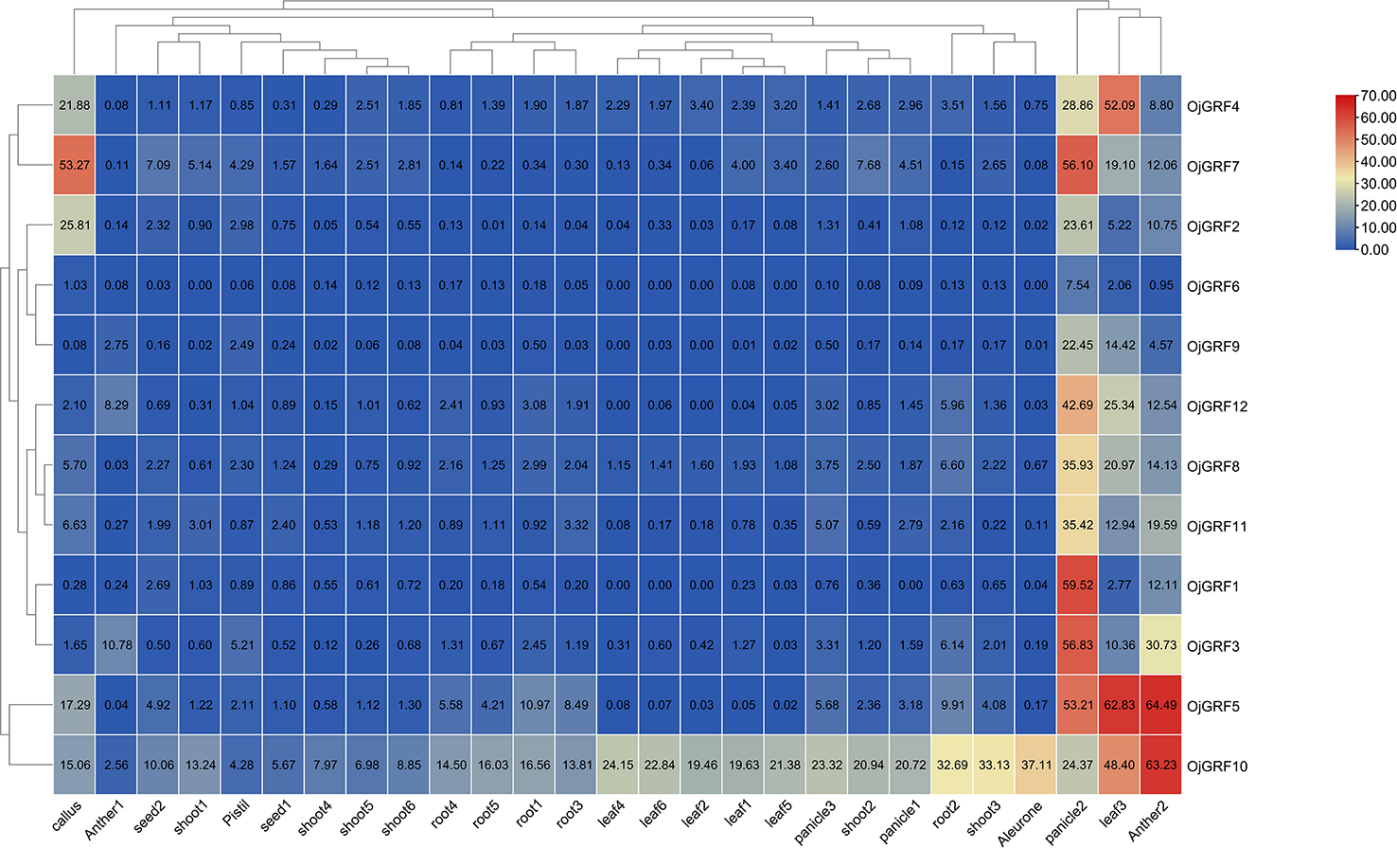
Expression profiles of the *GRFs* in the various rice tissues at different developmental stages. The heatmap based on the data of public rice transcriptomic by log_2_ FPKM represents the relative expression in 27 tissues. The *GRFs* are clustered according to hierarchical clustering. The blue and red color indicates the transcripts’ high and low expression levels, respectively.

To better understand the potential function of *GRF*s in rice, we analyzed public rice transcriptomic data (http://expression.ic4r.org/search) from different tissues, including the aleurone, antler, callus, leaf, panicle, pistil, root, seed and shoot, and found that the 12 *GRF*s ubiquitously expressed in rice (**Figure 9**), with particularly high expression in young inflorescence, implying that *GRF*s play an important role in inflorescence development (**Figure 9**). Additionally, *OjGRF4*, *OjGRF5*, and *OjGRF7* were highly expressed in leaf, anther, and callus, respectively, while *OjGRF10* was highly expressed throughout the growth period of rice (**Figure 9**), indicating the functional differentiation of *GRF*s in rice development.

**Figure 10:**
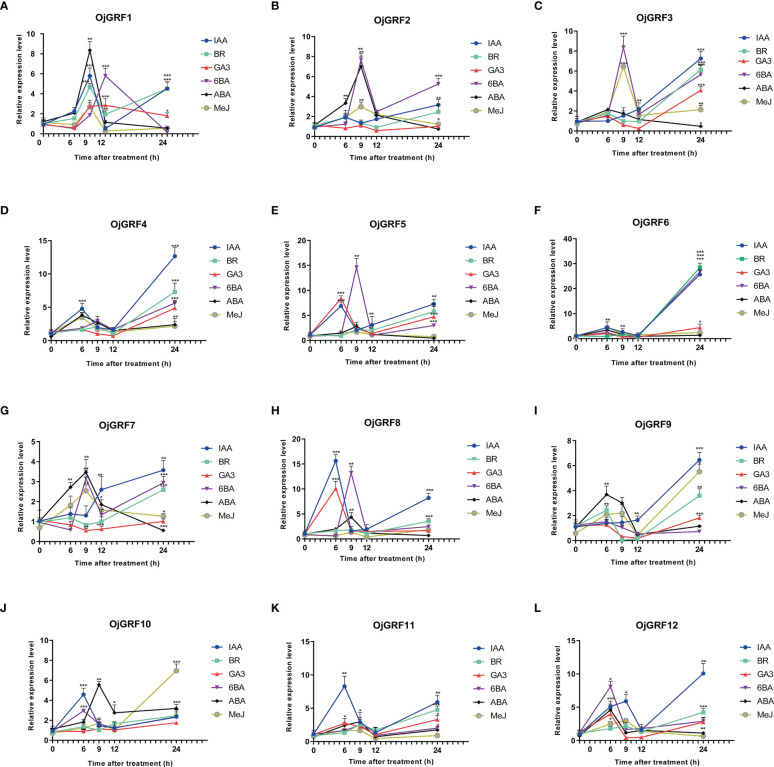
Analysis of the different expression patterns of *GRF* family members in rice under various hormonal treatments. **(A–L)** The expression of 12 *GRFs* in rice was calculated at 6, 9, 12, and 24 h of treatments compared with the expression value at 0h. The expression level of GRF was tested by qRT-PCR estimated by the 2^ΔΔCT^ method. The error bars show the standard deviation of the three biological replicates. Student’s t-test was used in this experiment; *p < 0.05; **p < 0.01; ***p < 0.001.

The analysis of cis-elements revealed that the *GRF* promoters contain a large number of cis-acting elements associated with ABA, IAA, MeJ, SA, and GA responses (**Figure 10**). Then, we investigated the expression of *GRF*s of rice under treatment of IAA, BR, GA3, 6BA, ABA, and MeJ. Results showed that all of the *GRF*s were significantly induced by the six hormones (**Figure 10**). Relatively, *OjGRF6 and OjGRF11* was most sensitive to IAA, BR, and ABA (**Figure 10F**), while *OjGRF3*, *OjGRF4*, and *OjGRF9* were most sensitive to IAA (**Figures 10C, D, I**). The expression of *OjGRF4* and *OjGRF6* showed a steep rise from the 6 h to the 24 h after hormone treatment, while the other ten *GRF*s had two apparent expression peaks after treatment (**Figures 10D, F**). *OjGRF1*, *OjGRF2*, and *OjGRF7* were induced to reach their highest after 9 h with ABA treatment, then began to decline (**Figures 10A, B, G**).

### *OjGRF11* affects root development *via* IAA pathway in rice

**Figure 11:**
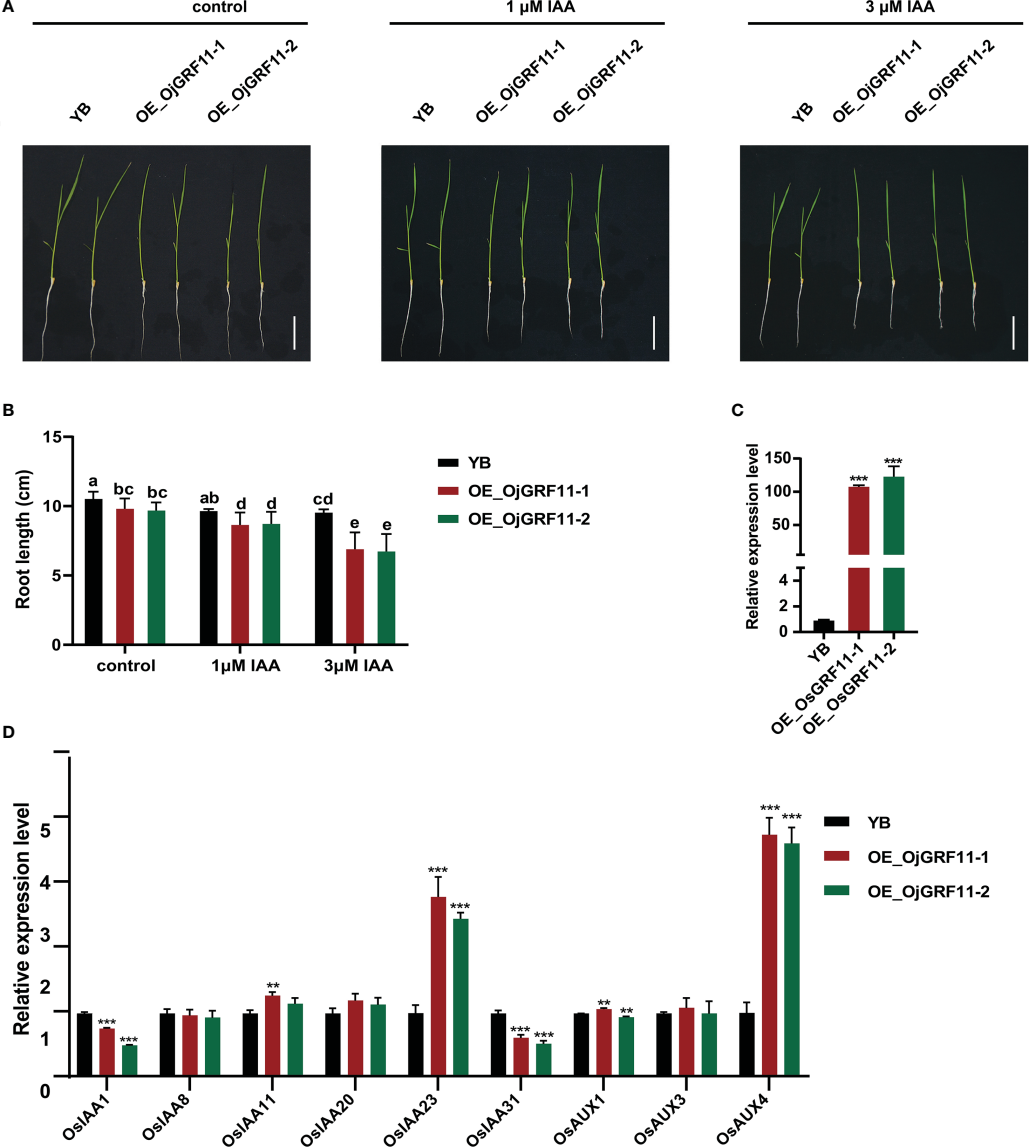
*OjGRF11* are insensitive to auxin treatment. **(A)** Characterization of root length in wild‐type (WT/YB) and *GRF11* over-expression lines under control, l μM IAA, and 3 μM IAA treatments for 7 d. Scale bars = 5 cm. **(B)** Statistical analysis of root length of in the WT/YB and *GRF11* overexpression transgenic plant under control, l μM IAA, and 3 μM IAA treatments for 7 d. The error bars show the standard deviation of the ten biological replicates. Different letters indicate significant differences (*P* < 0.05) determined by Duncan’s multiple range test. **(C)** Expression levels of *GRF11* in the WT/YB and *OjGRF11* overexpression transgenic plants. **(D)** Relative expression levels of the *OjGRF11*target genes in the transgenic plants compared with in the YB. In **(C, D)** the expression level was tested by qRT-PCR estimated by the 2^ΔΔCT^ method. The error bars show the standard deviation of the three biological replicates. ns: p > 0.05, **: p < 0.01, ***: p < 0.001.

The above results showed that *OjGRF11* was the one of earliest gene to responded to IAA treatment. Previous studies have shown that auxin signaling genes are involved in root development (Notaguchi et al., 2012; Yu et al., 2015; Cui et al., 2017; Ye et al., 2021). To investigate whether *OjGRF11* regulates root development through the IAA signaling pathway, an overexpression construct of *OjGRF11* was introduced into YB (*Oryza sativa L. ssp. indica*), and the *OjGRF11* transcript level was significantly increased, as assessed by qRT-PCR (**Figure 11C**). The statistical analysis of the root length of *OjGRF11* overexpression lines in 10-day-old seedlings showed that it was shorter than that of the WT/YB under hydroponic conditions (**Figures 11A, B**) indicating that *OjGRF11* negatively regulates root elongation.

To determine whether root length of *OjGRF11* over-expression lines respond to auxin treatment, these seedlings were grown in 0 μM IAA, 1 μM IAA, and 3 μM IAA nutrient solution. After 7 d of IAA treatment, the root length of *OjGRF11* over-expression lines was more suppressed than that of WT/YB (**Figures 11A, B**). Furthermore, an expression analysis verified that *OjGRF11* could be induced by an IAA treatment (**Supplementary Figure 3**). Thus, *OjGRF11*regulates the plants’ t root elongation.

To further confirm the effect of *OjGRF11* on root elongation through the IAA signal pathway, we examined several crucial genes in the IAA signaling pathway. The qRT-PCR results demonstrated that the expression levels of OsIAA23 and OsAUX4 were upregulated (**Figure 11D**). *OsIAA23* and *OsAUX4* are known to play critical roles in the development of root elongation (Jun et al., 2011; Ye et al., 2021). These findings indicate that *OjGRF11* is essential for regulating rice root development through the IAA pathway.

## Discussion

### Evolution of *GRFs* in the *Gramineae* crops

Previous results reflected that *GRFs* were specific in plant and play key roles in various plants growth and development (Fİlİz et al., 2014; Cao et al., 2016). Thus, it is necessary to systematically describe the evolutionary patterns of *GRFs* in different species. In this study, a total of 96 *GRF*s were identified in eight *Gramineae* crops (**Figure 1**, **Supplementary Table 2**), which were derived from two main types of gene duplication including tandem duplication and WGD/segmental duplication (**Table 1**), and the numbers of duplication gene pairs varied greatly among *Gramineae* crops (**Table 1**). However, except for two tandem duplicated gene pairs of *HvGRF2*/*HvGRF3* and *GRF4*/*GRF5* in *indica* rice (**Table 1**), the other paralogous *GRF*s were all homologs, they were derived from WGD/segmental duplication. Correspondingly, the GRFs had similar gene structure and function within the same category (**Figures 4**, **5**). For instance, the homologs *GRF4* in *indica* rice and *GRF5* in *japonica* rice were classified into Group 3 (**Figure 1**), which is known to be involved in auxiliary branch and spikelet development in rice (Gao et al., 2015). The 96 *GRF*s in *Gramineae* crops were categorized into three groups based on their structure, and each group showed some differences in terms of member composition, distribution of conserved motifs, and expression patterns (**Figure 4**), revealing the differentiation of *GRF*s in structure and function.

### Functional diversity in the *GRF* family

Transcription factors are considered to be key regulators of gene expression and coordinate the relationship between plants and the environment (Chen et al., 2021). Previous studies have demonstrated that *GRFs*, an important transcription factor in plants, is regulated by *miR396* (Liu et al., 2014; Gao et al., 2015; Omidbakhshfard et al., 2015). In our study, we have newly identified that *GRF5* in *japonica* rice is targeted by *miR166*, *miR395* and *miR6248* (**Supplementary Table 7**). Importantly, *miR166* and *miR395* have been implicated in cadmium stress response and disease resistance, respectively (Ding et al., 2018; Yang et al., 2022). Combined with the anoxic elements in the promoters of *OjGRF5* in *japonica* rice (**Figure 8**), it suggests that *GRF5* is possibly involved in both biological and abiotic stresses in rice.

Cis-acting elements prediction has been utilized in exploring gene function in various species (Cheng et al., 2021; Wang et al., 2021a; Wang et al., 2021b). In this study, a total of 41 cis-acting elements were identified in the *GRF* promoters. G-box (23.1%) and Box 4 (17.1%) occupied the largest number of light-responsive/responsiveness subcategories, which belong to the growth and development category (**Figure 8**). Recent reports indicate that *BnGRF2* regulates cell number and plant photosynthesis to enhance seed oil production in *Brassica napus* (Liu et al., 2012; Vercruyssen et al., 2015), and *AtGRF5* stimulates chloroplast division and photosynthesis in *Arabidopsis* (Liu et al., 2012; Vercruyssen et al., 2015). These findings suggest that *GRFs* are potentially involved in light-responsiveness and photosynthesis.

Increasing evidence shows that *GRF*s play critical roles in phytohormone response during plant growth and development (Knaap and Kende, 2000; Liu et al., 2014; Gao et al., 2015; Tang et al., 2018; Chen et al., 2020b). Our results showed that several motifs related to phytohormone response were identified (**Figure 8**), and all the *GRF*s in *japonica* rice were induced by IAA, BR, GA3, 6BA, ABA, and MeJ, although they showed various response patterns under different hormones (**Figure 10**). Notably, roots overexpressing *OjGRF11* were more sensitive to IAA (**Figure 11**). Furthermore, results showed that *OjGRF11* regulates root development by influencing genes in the auxin signaling pathway, particularly *OsIAA23* and *OsAUX4*, whose expression levels were significantly altered (**Figure 11**). However, further validation is needed to elucidate the molecular mechanisms underlying their interactions. Considering the high expression pattern of *GRFs* in young inflorescence, we can deduce that the *GRF*s are widely involved in the plant inflorescence development and responses to diverse environmental stresses in *Gramineae* crops.

## Conclusion

In this study, we found that the number and structure of *GRFs* in *Gramineae* crops were relatively stable and highly conserved. These proteins were highly expressed in the leaf and young inflorescence and contained rich cis-elements related to hormone response in their promoters. Furthermore, we observed that the expression of *GRFs* was induced under multiple hormonal stresses, including IAA, BR, GA3, 6BA, ABA, and MeJ treatments. Our investigation of *OjGRF11* revealed that this gene is significantly induced after 6 hours of IAA treatment and overexpression of this gene increases sensitivity to IAA and affects root elongation. These findings provide a comprehensive understanding of the molecular characteristics and evolutionary patterns of the GRF family in *Gramineae* crops.

## Data availability statement

The original contributions presented in the study are included in the article/**Supplementary Material**. Further inquiries can be directed to the corresponding authors.

## Author contributions

WW and HY designed the research; WW, MC carried out the bioinformatic analyses. WW performed subcellular localization and partial quantitative PCR experiments. XW, RW, FF and ZW participated in other molecular experiments; MC, WW and HY wrote the manuscript. ZT, SL and HY was responsible for revising the manuscript. All authors have read and agreed to the published version of the manuscript.
