# Corrigendum: Comprehensive evolutionary analysis of growth-regulating factor gene family revealing the potential molecular basis under multiple hormonal stress in *Gramineae* crops

**DOI:** 10.3389/fpls.2023.1222765

**Published:** 2023-05-30

**Authors:** Wei Wang, Mingxing Cheng, Xiao Wei, Ruihua Wang, Fengfeng Fan, Zhikai Wang, Zhihong Tian, Shaoqing Li, Huanran Yuan

**Affiliations:** ^1^ State Key Laboratory of Hybrid Rice, Key Laboratory for Research and Utilization of Heterosis in Indica Rice of Ministry of Agriculture, Engineering Research Center for Plant Biotechnology and Germplasm Utilization of Ministry of Education, College of Life Sciences, Wuhan University, Wuhan, China; ^2^ Hubei Hongshan Laboratory, Wuhan, China; ^3^ College of Life Science, Yangtze University, Jingzhou, China

**Keywords:** GRFs, *Gramineae* crops, evolutionary analysis, transcriptional activators, hormone treatments

In the published article, an author name was incorrectly written as **Huanan Yuan**. The correct spelling is **Huanran Yuan**.

The authors apologize for this error and state that this does not change the scientific conclusions of the article in any way. The original article has been updated.

