# Supplementary material for: Comprehensive evolutionary analysis of growth-regulating factor gene family revealing the potential molecular basis under multiple hormonal stress in Gramineae crops
Source: Front Plant Sci. 2023 Mar 31;14:1174955. doi: 10.3389/fpls.2023.1174955 (PMC10102486; doi:10.3389/fpls.2023.1174955)
Supplement: Supplementary file 1 [file DataSheet_1.docx]

***Supplementary Material***

**Comprehensive Evolutionary Analysis of Growth-regulating Factor Gene Family Revealing the Potential Molecular Basis under Multiple Hormonal Stress in Gramineae Crops**

Wei Wang^†1^, Mingxing Cheng^†1^, Xiao Wei^1^, Ruihua Wang^1^, Fengfeng Fan^1^, Zhikai Wang^2^, Zhihong Tian^2^, Shaoqing Li^*1^, Huanran Yuan^*1^

^1^State Key Laboratory of Hybrid Rice, Hongshan Laboratory of Hubei Province, Key Laboratory for Research and Utilization of Heterosis in Indica Rice of Ministry of Agriculture, Engineering Research Center for Plant Biotechnology and Germplasm Utilization of Ministry of Education, College of Life Science, Wuhan University, Wuhan 430072, China;

^2^ College of Life Science, Yangtze University, Jingzhou 434025, China;

*Corresponding author:

Huanran Yuan

Email: huanranyuan@whu.edu.cn

Shaoqing Li

Email: shaoqingli@whu.edu.cn

† These authors contributed equally to this work


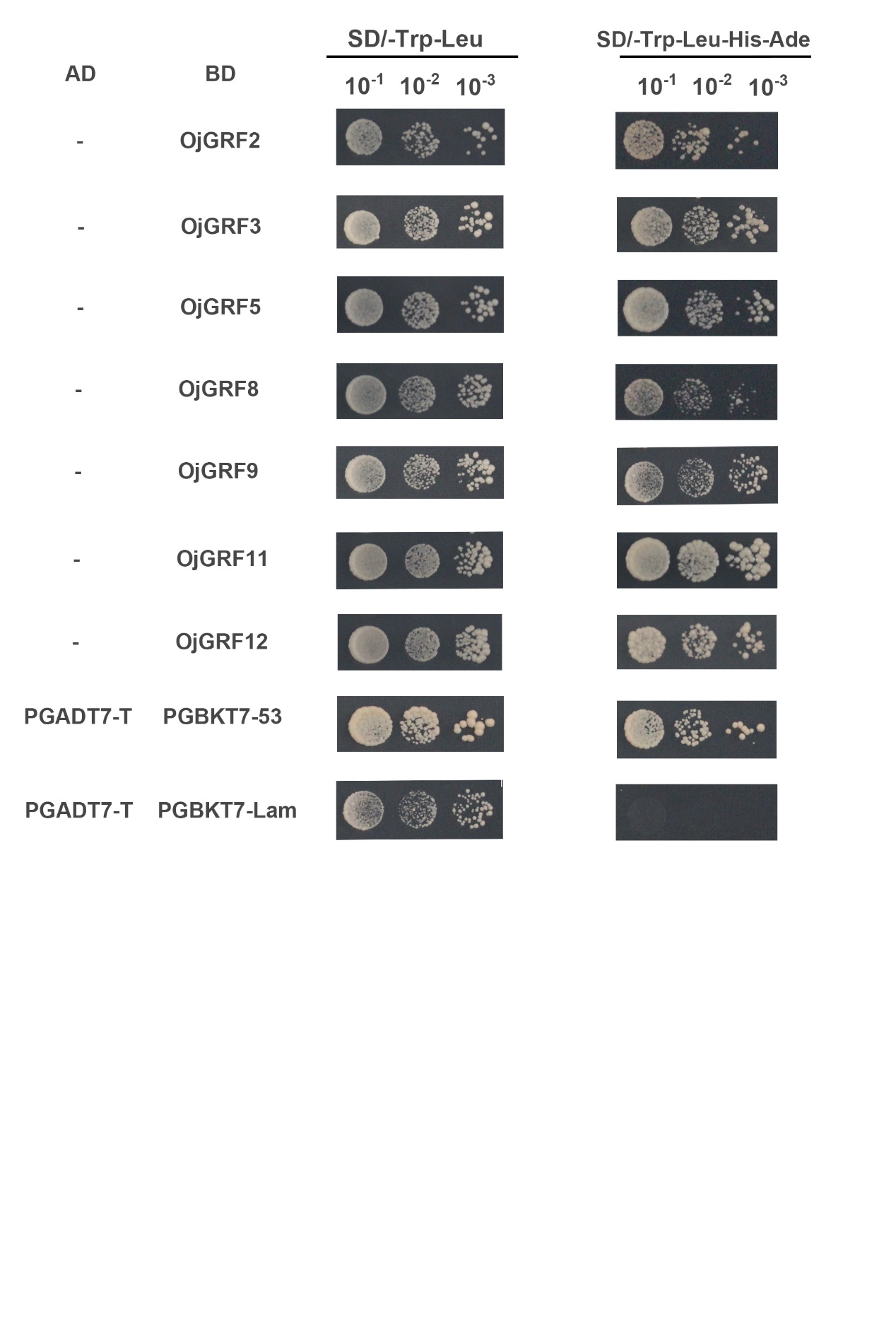


**Supplementary Figure 1.** Analysis of GRFs protein transcriptional self-activating activity in yeast.


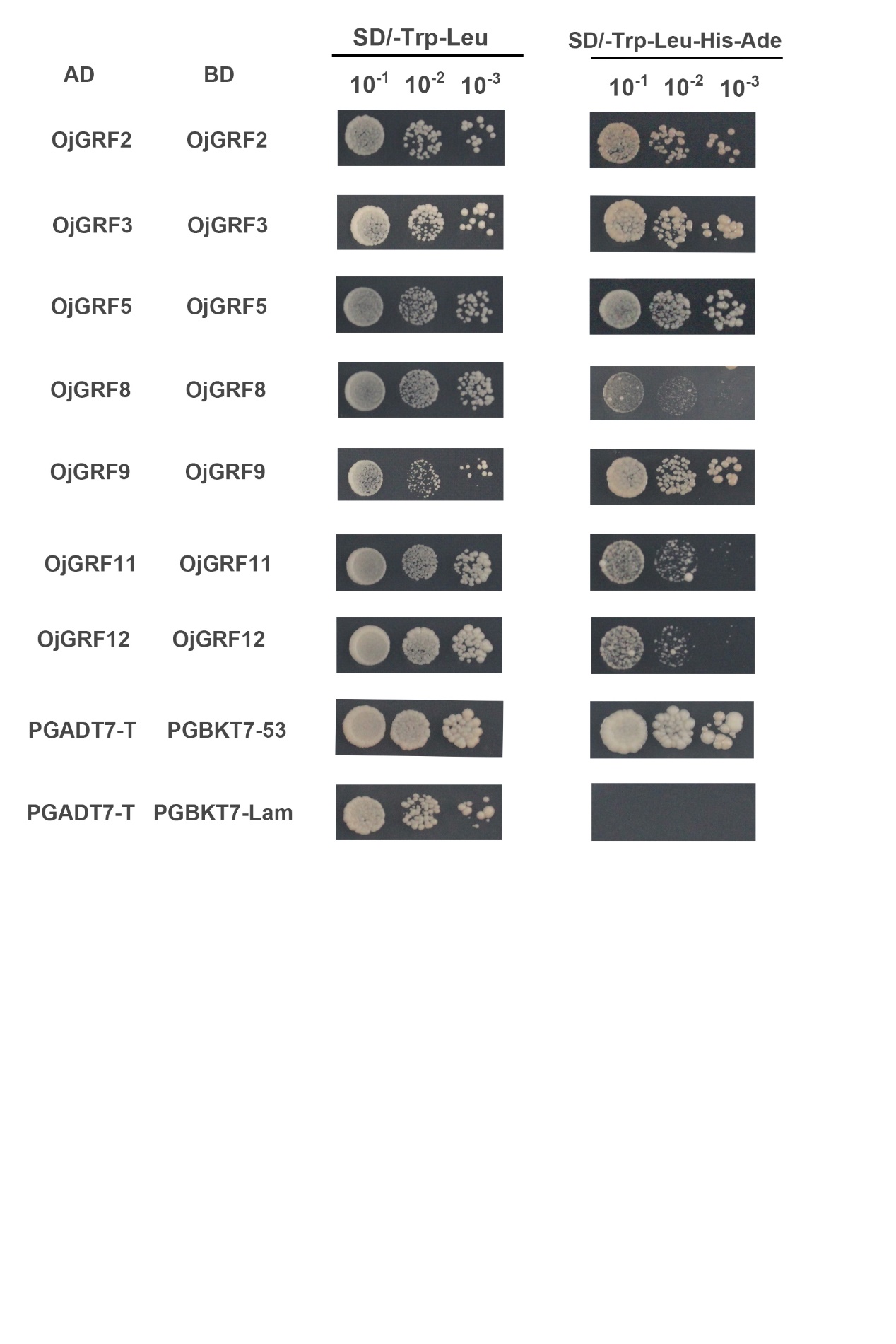


**Supplementary Figure 2.** Analysis of GRFs protein dimers in yeast.


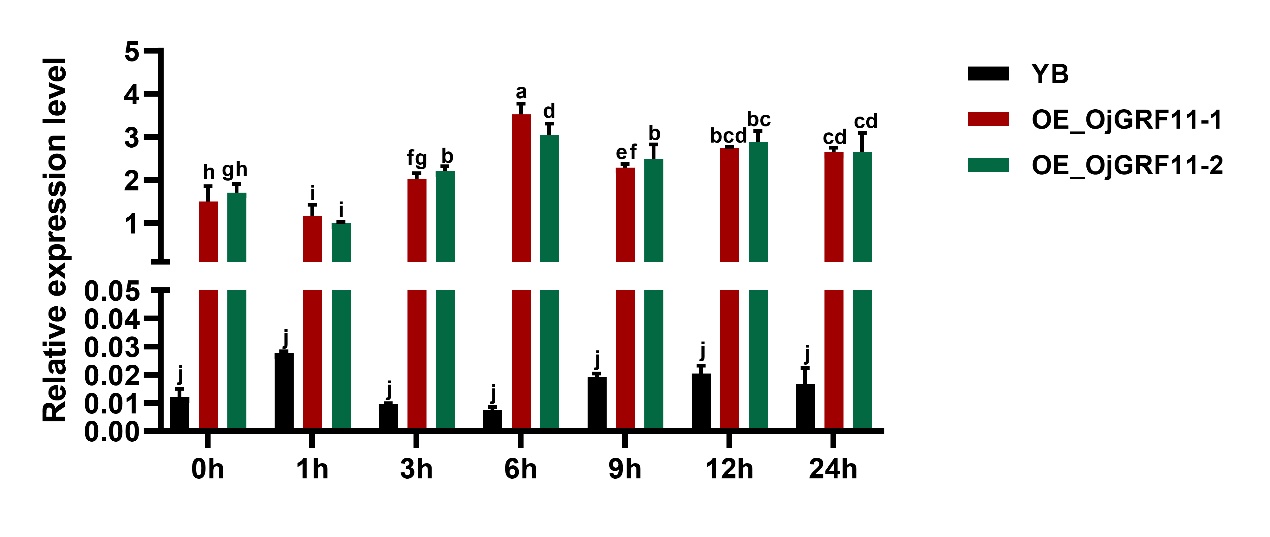


**Supplementary Figure 3.** qRT–PCR analysis of the *OjGRF11* transcripts after IAA treatments. The expression level of *GRF11* was tested by qRT-PCR estimated by the 2^∆∆CT^ method. The error bars show the standard deviation of the three biological replicates. Duncan’s multiple range test was used in this experiment; different letters indicate significant differences (*P* < 0.05

**Supplementary Table 1** The entry ID for all genes discussed in this work

| Species | Gene names | Entry ID | CHR | CHR.start | CHR.end |
| --- | --- | --- | --- | --- | --- |
| Brachypodium distachyon | BdGRF1 | Bradi1g09900.2 | Bd1 | 7118441 | 7122596 |
|  | BdGRF2 | Bradi1g12650.1 | Bd1 | 9517993 | 9520933 |
|  | BdGRF3 | Bradi1g28400.1 | Bd1 | 23655566 | 23659760 |
|  | BdGRF4 | Bradi1g46427.1 | Bd1 | 45111961 | 45114140 |
|  | BdGRF5 | Bradi1g50597.1 | Bd1 | 49354677 | 49358099 |
|  | BdGRF6 | Bradi2g14320.1 | Bd2 | 12901495 | 12905553 |
|  | BdGRF7 | Bradi3g51685.1 | Bd3 | 52490595 | 52491925 |
|  | BdGRF8 | Bradi3g52547.1 | Bd3 | 53200727 | 53204529 |
|  | BdGRF9 | Bradi3g57267.1 | Bd3 | 56693927 | 56696268 |
|  | BdGRF10 | Bradi4g16450.2 | Bd4 | 17224761 | 17228352 |
|  | BdGRF11 | Bradi5g18961.1 | Bd5 | 22197423 | 22199360 |
|  | BdGRF12 | Bradi5g20607.1 | Bd5 | 23598622 | 23602803 |
| Hordeum vulgare | HvGRF1 | HORVU2Hr1G054270.3 | chr2H | 332787218 | 332789588 |
|  | HvGRF2 | HORVU2Hr1G094460.2 | chr2H | 664885406 | 664887892 |
|  | HvGRF3 | HORVU2Hr1G094470.2 | chr2H | 664890347 | 664891461 |
|  | HvGRF4 | HORVU2Hr1G101770.7 | chr2H | 694689781 | 694693579 |
|  | HvGRF5 | HORVU4Hr1G003440.12 | chr4H | 7582926 | 7589204 |
|  | HvGRF6 | HORVU4Hr1G010080.6 | chr4H | 29847823 | 29851454 |
|  | HvGRF7 | HORVU4Hr1G037480.7 | chr4H | 280246683 | 280268392 |
|  | HvGRF8 | HORVU6Hr1G035720.2 | chr6H | 174063621 | 174064814 |
|  | HvGRF9 | HORVU6Hr1G065740.4 | chr6H | 453128633 | 453129616 |
|  | HvGRF10 | HORVU6Hr1G068370.1 | chr6H | 473135773 | 473139348 |
|  | HvGRF11 | HORVU6Hr1G081210.1 | chr6H | 544655166 | 544657178 |
|  | HvGRF12 | HORVU7Hr1G034610.4 | chr7H | 72772516 | 72773588 |
|  | HvGRF13 | HORVU0Hr1G016610.1 | chrUn | 89561655 | 89564811 |
| Oryza sativa subsp. indica | OiGRF1 | BGIOSGA008811-PA | chr2 | 29532907 | 29533751 |
|  | OiGRF2 | BGIOSGA005785-PA | chr2 | 30717538 | 30720921 |
|  | OiGRF3 | BGIOSGA005497-PA | chr2 | 34946498 | 34947899 |
|  | OiGRF4 | BGIOSGA013306-PA | chr3 | 30642152 | 30644584 |
|  | OiGRF5 | BGIOSGA013307-PA | chr3 | 30662209 | 30664632 |
|  | OiGRF6 | BGIOSGA013499-PA | chr3 | 34009047 | 34018002 |
|  | OiGRF7 | BGIOSGA016961-PA | chr4 | 27760521 | 27761477 |
|  | OiGRF8 | BGIOSGA014417-PA | chr4 | 29186737 | 29190301 |
|  | OiGRF9 | BGIOSGA022056-PA | chr6 | 1065372 | 1067990 |
|  | OiGRF10 | BGIOSGA022506-PA | chr6 | 6136033 | 6137409 |
|  | OiGRF11 | BGIOSGA025662-PA | chr7 | 15083382 | 15085357 |
|  | OiGRF12 | BGIOSGA035433-PA | chr11 | 17237151 | 17240143 |
|  | OiGRF13 | BGIOSGA037400-PA | chr12 | 14302485 | 14305820 |
| Oryza sativa subsp. japonica | OjGRF1 | LOC_Os02g45570.1 | Chr2 | 27727857 | 27728982 |
|  | OjGRF2 | LOC_Os02g47280.1 | Chr2 | 28863173 | 28866997 |
|  | OjGRF3 | LOC_Os02g53690.1 | Chr2 | 32840362 | 32842456 |
|  | OjGRF4 | LOC_Os03g47140.1 | Chr3 | 26679039 | 26682188 |
|  | OjGRF5 | LOC_Os03g51970.1 | Chr3 | 29823449 | 29825818 |
|  | OjGRF6 | LOC_Os04g48510.1 | Chr4 | 28921895 | 28923696 |
|  | OjGRF7 | LOC_Os04g51190.1 | Chr4 | 30317702 | 30321649 |
|  | OjGRF8 | LOC_Os06g02560.1 | Chr6 | 893172 | 896356 |
|  | OjGRF9 | LOC_Os06g10310.1 | Chr6 | 5300136 | 5301473 |
|  | OjGRF10 | LOC_Os07g28430.1 | Chr7 | 16622547 | 16625292 |
|  | OjGRF11 | LOC_Os11g35030.1 | Chr11 | 20521765 | 20525236 |
|  | OjGRF12 | LOC_Os12g29980.1 | Chr12 | 17915643 | 17919555 |
| Oryza rufipogon | OrGRF1 | ORUFI02G29340.1 | chr2 | 25930268 | 25931223 |
|  | OrGRF2 | ORUFI02G30740.1 | chr2 | 26975472 | 26978855 |
|  | OrGRF3 | ORUFI02G36060.1 | chr2 | 30940039 | 30941477 |
|  | OrGRF4 | ORUFI03G30390.1 | chr3 | 25197095 | 25199790 |
|  | OrGRF5 | ORUFI03G34250.1 | chr3 | 28278748 | 28282651 |
|  | OrGRF6 | ORUFI04G24060.1 | chr4 | 24469325 | 24471126 |
|  | OrGRF7 | ORUFI04G25800.1 | chr4 | 25774323 | 25777880 |
|  | OrGRF8 | ORUFI06G01080.1 | chr6 | 875792 | 878467 |
|  | OrGRF9 | ORUFI06G06870.1 | chr6 | 4821378 | 4822703 |
|  | OrGRF10 | ORUFI07G13880.1 | chr7 | 14275647 | 14278561 |
|  | OrGRF11 | ORUFI11G17860.2 | chr11 | 20622269 | 20625904 |
|  | OrGRF12 | ORUFI12G13380.1 | chr12 | 15021471 | 15025337 |
| Sorghum bicolor | SbGRF1 | Sobic.001G104500.1 | Chr01 | 8001993 | 8006115 |
|  | SbGRF2 | Sobic.001G139800.1 | Chr01 | 11114461 | 11117281 |
|  | SbGRF3 | Sobic.002G297800.1 | Chr02 | 67394510 | 67397153 |
|  | SbGRF4 | Sobic.004G269900.2 | Chr04 | 61417220 | 61421827 |
|  | SbGRF5 | Sobic.004G317000.1 | Chr04 | 65266845 | 65268995 |
|  | SbGRF6 | Sobic.005G150900.1 | Chr05 | 61984980 | 61988110 |
|  | SbGRF7 | Sobic.006G203400.1 | Chr06 | 55407501 | 55411315 |
|  | SbGRF8 | Sobic.010G077200.1 | Chr10 | 6374926 | 6376491 |
| Setaria italica | SiGRF1 | Seita.1G271800.1 | scaffold_1 | 34496412 | 34497672 |
|  | SiGRF2 | Seita.1G287100.1 | scaffold_1 | 35670427 | 35674285 |
|  | SiGRF3 | Seita.1G338700.1 | scaffold_1 | 39560848 | 39562762 |
|  | SiGRF4 | Seita.2G309100.1 | scaffold_2 | 40019405 | 40021928 |
|  | SiGRF5 | Seita.4G012400.1 | scaffold_4 | 772532 | 775361 |
|  | SiGRF6 | Seita.4G086400.1 | scaffold_4 | 7065794 | 7067627 |
|  | SiGRF7 | Seita.7G224500.1 | scaffold_7 | 28997252 | 29001151 |
|  | SiGRF8 | Seita.8G149500.1 | scaffold_8 | 28682967 | 28686754 |
|  | SiGRF9 | Seita.9G107700.1 | scaffold_9 | 6516669 | 6520803 |
|  | SiGRF10 | Seita.9G141900.1 | scaffold_9 | 9066531 | 9071706 |
| Zea mays | ZmGRF1 | Zm00001d033396_T003 | chr1 | 261791305 | 261794041 |
|  | ZmGRF2 | Zm00001d033876_T004 | chr1 | 277130909 | 277134992 |
|  | ZmGRF3 | Zm00001d002429_T003 | chr2 | 12492600 | 12496031 |
|  | ZmGRF4 | Zm00001d006348_T001 | chr2 | 205258531 | 205260875 |
|  | ZmGRF5 | Zm00001d007465_T001 | chr2 | 232275513 | 232278071 |
|  | ZmGRF6 | Zm00001d051456_T001 | chr4 | 159322444 | 159323547 |
|  | ZmGRF7 | Zm00001d052112_T001 | chr4 | 180147356 | 180150053 |
|  | ZmGRF8 | Zm00001d013346_T001 | chr5 | 9051468 | 9063110 |
|  | ZmGRF9 | Zm00001d013555_T001 | chr5 | 14173339 | 14176252 |
|  | ZmGRF10 | Zm00001d017742_T001 | chr5 | 205734245 | 205737674 |
|  | ZmGRF11 | Zm00001d018260_T001 | chr5 | 217546954 | 217548912 |
|  | ZmGRF12 | Zm00001d035965_T004 | chr6 | 62968987 | 62972013 |
|  | ZmGRF13 | Zm00001d037117_T001 | chr6 | 112965440 | 112967072 |
|  | ZmGRF14 | Zm00001d021362_T001 | chr7 | 149911394 | 149914056 |
|  | ZmGRF15 | Zm00001d045533_T001 | chr9 | 25739313 | 25742304 |
|  | ZmGRF16 | Zm00001d026240_T006 | chr10 | 141714630 | 141718409 |

This work identified 96 GRF gene family members in eight Gramineae crops. The gene names, entry ID, CHR, CHR.start and CHR.end of different species were listed.

**Supplementary Table 2** The number of three clades among eight species

| Species | Group1 | Group2 | | Group3 | Total | Genome size |  |
| --- | --- | --- | --- | --- | --- | --- | --- |
| Brachypodium_distachyon | | 4 | | 5 | 3 | 12 | 355 Mbp |
| Hordeum_vulgare | | 4 | | 5 | 4 | 13 | 4.83 Gbp |
| Oryza_sativa_subsp_indica | | 3 | | 5 | 5 | 13 | 427 Mbp |
| Oryza_sativa_ssp_japonica | | 3 | | 5 | 4 | 12 | 382 Mbp |
| Oryza_rufipogon | | 3 | | 5 | 4 | 12 | 339 Mbp |
| Sorghum_bicolor | | 1 | | 4 | 3 | 8 | 730 Mbp |
| Setaria_italica | | 2 | | 5 | 3 | 10 | 490 Mbp |
| Zea_mays | | 3 | | 8 | 5 | 16 | 2.4 Gbp |
| Total | | 23 | | 42 | 31 | 96 |  |

A total of 96 *GRF* genes, including 12, 13, 13, 12, 12, 8, 10 and 16 *GRF* genes were identified in eight species, respectively. The GRF proteins were classified into three cluding Clade 1, Clade 2, Clade 3.

**Supplementary Table 3** The orthologous gene pairs among test *Gramineae* crop

|  | Bd | Hv | Oi | Oj | Or | Sb | Si | Zm | Total |
| --- | --- | --- | --- | --- | --- | --- | --- | --- | --- |
| OG00 | 5 | 5 | 5 | 5 | 5 | 4 | 5 | 8 | 42 |
| OG01 | 4 | 2 | 3 | 3 | 3 | 1 | 2 | 3 | 21 |
| OG02 | 2 | 2 | 3 | 3 | 3 | 2 | 2 | 3 | 20 |
| OG03 | 1 | 1 | 2 | 1 | 1 | 1 | 1 | 2 | 10 |
| OG04 | 0 | 3 | 0 | 0 | 0 | 0 | 0 | 0 | 3 |
| Total | 12 | 13 | 13 | 12 | 12 | 8 | 10 | 16 | 96 |

The orthologous groups (OGs) were identified and 96 genes were divided into 5 OGs.

**Supplementary Table 4** The *GRFs* in each orthogroup

| Orthogroup | OG00 | OG01 | OG02 | OG03 | OG04 |
| --- | --- | --- | --- | --- | --- |
| BdGRF | BdGRF12, BdGRF4, BdGRF5, BdGRF8, BdGRF9 | BdGRF11, BdGRF3, BdGRF6, BdGRF7 | BdGRF1, BdGRF10 | BdGRF2 |  |
| HvGRF | HvGRF10, HvGRF11, HvGRF12, HvGRF13, HvGRF4 | HvGRF1, HvGRF9 | HvGRF6, HvGRF7 | HvGRF5 | HvGRF2, HvGRF3, HvGRF8 |
| OiGRF | OiGRF10, OiGRF2, OiGRF3, OiGRF8, OiGRF9 | OiGRF1, OiGRF11, OiGRF7 | OiGRF12, OiGRF13, OiGRF6 | OiGRF4, OiGRF5 |  |
| OjGRF | OjGRF2, OjGRF3, OjGRF7, OjGRF8, OjGRF9 | OjGRF1, OjGRF10, OjGRF6 | OjGRF11, OjGRF12, OjGRF5 | OjGRF4 |  |
| OrGRF | OrGRF2, OrGRF3, OrGRF7, OrGRF8, OrGRF9 | OrGRF1, OrGRF10, OrGRF6 | OrGRF11, OrGRF12, OrGRF5 | OrGRF4 |  |
| SbGRF | SbGRF4, SbGRF5, SbGRF7, SbGRF8 | SbGRF3 | SbGRF1, SbGRF6 | SbGRF2 |  |
| SiGRF | SiGRF2, SiGRF3, SiGRF5, SiGRF6, SiGRF7 | SiGRF1, SiGRF4 | SiGRF8, SiGRF9 | SiGRF10 |  |
| ZmGRF | ZmGRF10, ZmGRF11, ZmGRF12, ZmGRF13, ZmGRF15, ZmGRF16, ZmGRF3, ZmGRF7 | ZmGRF14, ZmGRF4, ZmGRF6 | ZmGRF2, ZmGRF5, ZmGRF8 | ZmGRF1, ZmGRF9 |  |

**Supplementary Table 5** Secondary structure of *GRF* transcription factors family members in rice

| Gene name | Alpha helix | | Extended strand | | Beta turn | | Random coil | |
| --- | --- | --- | --- | --- | --- | --- | --- | --- |
|  | number | Proportion (%) | number | Proportion (%) | number | Proportion (%) | number | Proportion (%) |
| OjGRF1 | 63 | 29.86% | 19 | 9.00% | 8 | 3.79% | 121 | 57.35% |
| OjGRF2 | 83 | 19.67% | 30 | 7.11% | 13 | 3.08% | 296 | 70.14% |
| OjGRF3 | 95 | 23.93% | 49 | 12.34% | 25 | 6.30% | 228 | 57.43% |
| OjGRF4 | 118 | 27.70% | 39 | 9.15% | 20 | 4.69% | 249 | 58.45% |
| OjGRF5 | 91 | 19.96% | 48 | 10.53% | 9 | 1.97% | 308 | 67.54% |
| OjGRF6 | 46 | 19.49% | 35 | 14.83% | 17 | 7.20% | 138 | 58.47% |
| OjGRF7 | 83 | 21.45% | 20 | 5.17% | 12 | 3.10% | 272 | 70.28% |
| OjGRF8 | 49 | 13.76% | 39 | 10.96% | 16 | 4.49% | 252 | 70.79% |
| OjGRF9 | 87 | 28.90% | 15 | 4.98% | 9 | 2.99% | 190 | 63.12% |
| OjGRF10 | 90 | 33.46% | 32 | 11.90% | 8 | 2.97% | 139 | 51.67% |
| OjGRF11 | 78 | 19.07% | 55 | 13.45% | 27 | 6.60% | 249 | 60.88% |
| OjGRF12 | 85 | 19.77% | 48 | 11.16% | 15 | 3.49% | 282 | 65.58% |

**Supplementary Table 6** The nuclear localization signal prediction of GRF in rice

| Gene_id | Location | Prediction sequence |
| --- | --- | --- |
| OjGRF1 | 146-177 | GKKWRCSRDVVPGHKYCERHVHRGRGRSRKPV |
| OjGRF2 | 10-38, 160-194 | TTARPRQPRPPPSSPSAAPPRSPRKQREP, RRTDGKKWRCSKEAAPDSKYCERHMHRGRNRSRKP |
| OjGRF3 | 97-131 | RRTDGKKWRCSKEAYPDSKYCEKHMHRGKNRSRKP |
| OjGRF4 | 179-200 | ERHINRNRHRSRKPVENQSRKT |
| OjGRF5 | 101-125 | KYCERHMNRGRHRSRKHVEGQPGHA |
| OjGRF6 | 151-182 | GKKWRCSRDVVPGHKYCERHVHRGRGRSRKPM |
| OjGRF7 | 121-155 | RRTDGKKWRCSKEAAPDSKYCERHMHRGRNRSRKP |
| OjGRF8 | 108-142 | GKKWRCSKEAHPDSKYCERHMHRGRNRSRKPVESK |
| OjGRF9 | 98-130 | GKKWRCSREAYGESKYCEKHMHRGKNRSRKPVE |
| OjGRF10 | 207-219, 235-247 | CERHMHRGRKRPV, KPASGKATEGGKK |
| OjGRF11 | 166-205 | RTDGKKWRCSRDAVGDQKYCERHINRGRHRSRKHVEGRKA |
| OjGRF12 | 154-169 | CERHINRGRHRSRKHV |

**Supplementary Table 7** Predict the miRNAs target sites of *GRF* genes

| miRNA_Acc. | Target_Acc. | Target_start | Target_end | miRNA_aligned_fragment | Target_aligned_fragment | Inhibition |
| --- | --- | --- | --- | --- | --- | --- |
| osa-miR396d | OjGRF8 | 399 | 419 | UCCACAGGCUUUCUUGAACGG | CCGUUCAAGAAAGCCUGUGGA | Cleavage |
| osa-miR396d | OjGRF7 | 450 | 470 | UCCACAGGCUUUCUUGAACGG | CCGUUCAAGAAAGCCUGUGGA | Cleavage |
| osa-miR396d | OjGRF4 | 561 | 581 | UCCACAGGCUUUCUUGAACGG | CCGUUCAAGAAAGCCUGUGGA | Cleavage |
| osa-miR396d | OjGRF2 | 567 | 587 | UCCACAGGCUUUCUUGAACGG | CCGUUCAAGAAAGCCUGUGGA | Cleavage |
| osa-miR396d | OjGRF3 | 378 | 398 | UCCACAGGCUUUCUUGAACGG | CCGUUCAAGAAAGCCUGUGGA | Cleavage |
| osa-miR396d | OjGRF1 | 513 | 533 | UCCACAGGCUUUCUUGAACGG | CCGUUCAAGAAAGCCUGUGGA | Cleavage |
| osa-miR396d | OjGRF9 | 369 | 389 | UCCACAGGCUUUCUUGAACGG | CCGUUCAAGAAAGCCUGUGGA | Cleavage |
| osa-miR396e-5p | OjGRF4 | 561 | 581 | UCCACAGGCUUUCUUGAACUG | CCGUUCAAGAAAGCCUGUGGA | Cleavage |
| osa-miR396e-5p | OjGRF8 | 399 | 419 | UCCACAGGCUUUCUUGAACUG | CCGUUCAAGAAAGCCUGUGGA | Cleavage |
| osa-miR396e-5p | OjGRF1 | 513 | 533 | UCCACAGGCUUUCUUGAACUG | CCGUUCAAGAAAGCCUGUGGA | Cleavage |
| osa-miR396e-5p | OjGRF2 | 567 | 587 | UCCACAGGCUUUCUUGAACUG | CCGUUCAAGAAAGCCUGUGGA | Cleavage |
| osa-miR396e-5p | OjGRF3 | 378 | 398 | UCCACAGGCUUUCUUGAACUG | CCGUUCAAGAAAGCCUGUGGA | Cleavage |
| osa-miR396e-5p | OjGRF7 | 450 | 470 | UCCACAGGCUUUCUUGAACUG | CCGUUCAAGAAAGCCUGUGGA | Cleavage |
| osa-miR396e-5p | OjGRF9 | 369 | 389 | UCCACAGGCUUUCUUGAACUG | CCGUUCAAGAAAGCCUGUGGA | Cleavage |
| osa-miR396g | OjGRF9 | 369 | 389 | UCCACAGGCUUUCUUGAACGG | CCGUUCAAGAAAGCCUGUGGA | Cleavage |
| osa-miR396g | OjGRF8 | 399 | 419 | UCCACAGGCUUUCUUGAACGG | CCGUUCAAGAAAGCCUGUGGA | Cleavage |
| osa-miR396g | OjGRF4 | 561 | 581 | UCCACAGGCUUUCUUGAACGG | CCGUUCAAGAAAGCCUGUGGA | Cleavage |
| osa-miR396g | OjGRF7 | 450 | 470 | UCCACAGGCUUUCUUGAACGG | CCGUUCAAGAAAGCCUGUGGA | Cleavage |
| osa-miR396g | OjGRF1 | 513 | 533 | UCCACAGGCUUUCUUGAACGG | CCGUUCAAGAAAGCCUGUGGA | Cleavage |
| osa-miR396g | OjGRF2 | 567 | 587 | UCCACAGGCUUUCUUGAACGG | CCGUUCAAGAAAGCCUGUGGA | Cleavage |
| osa-miR396g | OjGRF3 | 378 | 398 | UCCACAGGCUUUCUUGAACGG | CCGUUCAAGAAAGCCUGUGGA | Cleavage |
| osa-miR396h | OjGRF3 | 378 | 398 | UCCACAGGCUUUCUUGAACGG | CCGUUCAAGAAAGCCUGUGGA | Cleavage |
| osa-miR396h | OjGRF9 | 369 | 389 | UCCACAGGCUUUCUUGAACGG | CCGUUCAAGAAAGCCUGUGGA | Cleavage |
| osa-miR396h | OjGRF4 | 561 | 581 | UCCACAGGCUUUCUUGAACGG | CCGUUCAAGAAAGCCUGUGGA | Cleavage |
| osa-miR396h | OjGRF2 | 567 | 587 | UCCACAGGCUUUCUUGAACGG | CCGUUCAAGAAAGCCUGUGGA | Cleavage |
| osa-miR396h | OjGRF7 | 450 | 470 | UCCACAGGCUUUCUUGAACGG | CCGUUCAAGAAAGCCUGUGGA | Cleavage |
| osa-miR396h | OjGRF8 | 399 | 419 | UCCACAGGCUUUCUUGAACGG | CCGUUCAAGAAAGCCUGUGGA | Cleavage |
| osa-miR396h | OjGRF1 | 513 | 533 | UCCACAGGCUUUCUUGAACGG | CCGUUCAAGAAAGCCUGUGGA | Cleavage |
| osa-miR396d | OjGRF5 | 336 | 356 | UCCACAGGCUUUCUUGAACGG | CCGUUCAAGAAAGCAUGUGGA | Cleavage |
| osa-miR396d | OjGRF12 | 489 | 509 | UCCACAGGCUUUCUUGAACGG | CCGUUCAAGAAAGCAUGUGGA | Cleavage |
| osa-miR396d | OjGRF6 | 528 | 548 | UCCACAGGCUUUCUUGAACGG | CCGUUCAAGAAAGCCUAUGGA | Cleavage |
| osa-miR396d | OjGRF11 | 582 | 602 | UCCACAGGCUUUCUUGAACGG | UCGUUCAAGAAAGCAUGUGGA | Cleavage |
| osa-miR396e-5p | OjGRF5 | 336 | 356 | UCCACAGGCUUUCUUGAACUG | CCGUUCAAGAAAGCAUGUGGA | Cleavage |
| osa-miR396e-5p | OjGRF12 | 489 | 509 | UCCACAGGCUUUCUUGAACUG | CCGUUCAAGAAAGCAUGUGGA | Cleavage |
| osa-miR396e-5p | OjGRF6 | 528 | 548 | UCCACAGGCUUUCUUGAACUG | CCGUUCAAGAAAGCCUAUGGA | Cleavage |
| osa-miR396e-5p | OjGRF11 | 582 | 602 | UCCACAGGCUUUCUUGAACUG | UCGUUCAAGAAAGCAUGUGGA | Cleavage |
| osa-miR396f-5p | OjGRF2 | 568 | 589 | UCUCCACAGGCUUUCUUGAACU | CGUUCAAGAAAGCCUGUGGAAA | Cleavage |
| osa-miR396f-5p | OjGRF3 | 379 | 400 | UCUCCACAGGCUUUCUUGAACU | CGUUCAAGAAAGCCUGUGGAAA | Cleavage |
| osa-miR396f-5p | OjGRF9 | 370 | 391 | UCUCCACAGGCUUUCUUGAACU | CGUUCAAGAAAGCCUGUGGAAA | Cleavage |
| osa-miR396f-5p | OjGRF4 | 562 | 583 | UCUCCACAGGCUUUCUUGAACU | CGUUCAAGAAAGCCUGUGGAAA | Cleavage |
| osa-miR396g | OjGRF6 | 528 | 548 | UCCACAGGCUUUCUUGAACGG | CCGUUCAAGAAAGCCUAUGGA | Cleavage |
| osa-miR396g | OjGRF5 | 336 | 356 | UCCACAGGCUUUCUUGAACGG | CCGUUCAAGAAAGCAUGUGGA | Cleavage |
| osa-miR396g | OjGRF12 | 489 | 509 | UCCACAGGCUUUCUUGAACGG | CCGUUCAAGAAAGCAUGUGGA | Cleavage |
| osa-miR396g | OjGRF11 | 582 | 602 | UCCACAGGCUUUCUUGAACGG | UCGUUCAAGAAAGCAUGUGGA | Cleavage |
| osa-miR396h | OjGRF6 | 528 | 548 | UCCACAGGCUUUCUUGAACGG | CCGUUCAAGAAAGCCUAUGGA | Cleavage |
| osa-miR396h | OjGRF5 | 336 | 356 | UCCACAGGCUUUCUUGAACGG | CCGUUCAAGAAAGCAUGUGGA | Cleavage |
| osa-miR396h | OjGRF12 | 489 | 509 | UCCACAGGCUUUCUUGAACGG | CCGUUCAAGAAAGCAUGUGGA | Cleavage |
| osa-miR396h | OjGRF11 | 582 | 602 | UCCACAGGCUUUCUUGAACGG | UCGUUCAAGAAAGCAUGUGGA | Cleavage |
| osa-miR2924 | OjGRF10 | 112 | 132 | CUCGCUUGCUCCGGCCGCCAC | GCGGCGGUCGGGGUGGGCGAG | Cleavage |
| osa-miR396f-5p | OjGRF8 | 400 | 421 | UCUCCACAGGCUUUCUUGAACU | CGUUCAAGAAAGCCUGUGGAAU | Cleavage |
| osa-miR396f-5p | OjGRF7 | 451 | 472 | UCUCCACAGGCUUUCUUGAACU | CGUUCAAGAAAGCCUGUGGAAG | Cleavage |
| osa-miR396f-5p | OjGRF1 | 514 | 535 | UCUCCACAGGCUUUCUUGAACU | CGUUCAAGAAAGCCUGUGGAAG | Cleavage |
| osa-miR167e-3p | OjGRF11 | 858 | 878 | AGAUCAUGUUGCAGCUUCACU | ACUGAAGUUGCACCAUGAUCA | Cleavage |
| osa-miR167i-3p | OjGRF11 | 858 | 878 | AGAUCAUGUUGCAGCUUCACU | ACUGAAGUUGCACCAUGAUCA | Cleavage |
| osa-miR396a-5p | OjGRF12 | 489 | 510 | UUCCACA-GCUUUCUUGAACUG | CCGUUCAAGAAAGCAUGUGGAA | Cleavage |
| osa-miR396a-5p | OjGRF8 | 399 | 420 | UUCCACA-GCUUUCUUGAACUG | CCGUUCAAGAAAGCCUGUGGAA | Cleavage |
| osa-miR396a-5p | OjGRF3 | 378 | 399 | UUCCACA-GCUUUCUUGAACUG | CCGUUCAAGAAAGCCUGUGGAA | Cleavage |
| osa-miR396a-5p | OjGRF7 | 450 | 471 | UUCCACA-GCUUUCUUGAACUG | CCGUUCAAGAAAGCCUGUGGAA | Cleavage |
| osa-miR396a-5p | OjGRF5 | 336 | 357 | UUCCACA-GCUUUCUUGAACUG | CCGUUCAAGAAAGCAUGUGGAA | Cleavage |
| osa-miR396a-5p | OjGRF1 | 513 | 534 | UUCCACA-GCUUUCUUGAACUG | CCGUUCAAGAAAGCCUGUGGAA | Cleavage |
| osa-miR396a-5p | OjGRF9 | 369 | 390 | UUCCACA-GCUUUCUUGAACUG | CCGUUCAAGAAAGCCUGUGGAA | Cleavage |
| osa-miR396a-5p | OjGRF4 | 561 | 582 | UUCCACA-GCUUUCUUGAACUG | CCGUUCAAGAAAGCCUGUGGAA | Cleavage |
| osa-miR396a-5p | OjGRF2 | 567 | 588 | UUCCACA-GCUUUCUUGAACUG | CCGUUCAAGAAAGCCUGUGGAA | Cleavage |
| osa-miR396a-5p | OjGRF11 | 582 | 603 | UUCCACA-GCUUUCUUGAACUG | UCGUUCAAGAAAGCAUGUGGAA | Cleavage |
| osa-miR396b-5p | OjGRF7 | 450 | 471 | UUCCACA-GCUUUCUUGAACUG | CCGUUCAAGAAAGCCUGUGGAA | Cleavage |
| osa-miR396b-5p | OjGRF3 | 378 | 399 | UUCCACA-GCUUUCUUGAACUG | CCGUUCAAGAAAGCCUGUGGAA | Cleavage |
| osa-miR396b-5p | OjGRF5 | 336 | 357 | UUCCACA-GCUUUCUUGAACUG | CCGUUCAAGAAAGCAUGUGGAA | Cleavage |
| osa-miR396b-5p | OjGRF8 | 399 | 420 | UUCCACA-GCUUUCUUGAACUG | CCGUUCAAGAAAGCCUGUGGAA | Cleavage |
| osa-miR396b-5p | OjGRF12 | 489 | 510 | UUCCACA-GCUUUCUUGAACUG | CCGUUCAAGAAAGCAUGUGGAA | Cleavage |
| osa-miR396b-5p | OjGRF1 | 513 | 534 | UUCCACA-GCUUUCUUGAACUG | CCGUUCAAGAAAGCCUGUGGAA | Cleavage |
| osa-miR396b-5p | OjGRF9 | 369 | 390 | UUCCACA-GCUUUCUUGAACUG | CCGUUCAAGAAAGCCUGUGGAA | Cleavage |
| osa-miR396b-5p | OjGRF2 | 567 | 588 | UUCCACA-GCUUUCUUGAACUG | CCGUUCAAGAAAGCCUGUGGAA | Cleavage |
| osa-miR396b-5p | OjGRF4 | 561 | 582 | UUCCACA-GCUUUCUUGAACUG | CCGUUCAAGAAAGCCUGUGGAA | Cleavage |
| osa-miR396b-5p | OjGRF11 | 582 | 603 | UUCCACA-GCUUUCUUGAACUG | UCGUUCAAGAAAGCAUGUGGAA | Cleavage |
| osa-miR396c-5p | OjGRF7 | 450 | 471 | UUCCACA-GCUUUCUUGAACUU | CCGUUCAAGAAAGCCUGUGGAA | Cleavage |
| osa-miR396c-5p | OjGRF4 | 561 | 582 | UUCCACA-GCUUUCUUGAACUU | CCGUUCAAGAAAGCCUGUGGAA | Cleavage |
| osa-miR396c-5p | OjGRF5 | 336 | 357 | UUCCACA-GCUUUCUUGAACUU | CCGUUCAAGAAAGCAUGUGGAA | Cleavage |
| osa-miR396c-5p | OjGRF12 | 489 | 510 | UUCCACA-GCUUUCUUGAACUU | CCGUUCAAGAAAGCAUGUGGAA | Cleavage |
| osa-miR396c-5p | OjGRF8 | 399 | 420 | UUCCACA-GCUUUCUUGAACUU | CCGUUCAAGAAAGCCUGUGGAA | Cleavage |
| osa-miR396c-5p | OjGRF1 | 513 | 534 | UUCCACA-GCUUUCUUGAACUU | CCGUUCAAGAAAGCCUGUGGAA | Cleavage |
| osa-miR396c-5p | OjGRF9 | 369 | 390 | UUCCACA-GCUUUCUUGAACUU | CCGUUCAAGAAAGCCUGUGGAA | Cleavage |
| osa-miR396c-5p | OjGRF2 | 567 | 588 | UUCCACA-GCUUUCUUGAACUU | CCGUUCAAGAAAGCCUGUGGAA | Cleavage |
| osa-miR396c-5p | OjGRF3 | 378 | 399 | UUCCACA-GCUUUCUUGAACUU | CCGUUCAAGAAAGCCUGUGGAA | Cleavage |
| osa-miR396c-5p | OjGRF11 | 582 | 603 | UUCCACA-GCUUUCUUGAACUU | UCGUUCAAGAAAGCAUGUGGAA | Cleavage |
| osa-miR167a-5p | OjGRF4 | 107 | 127 | UGAAGCUGCCAGCAUGAUCUA | GAGCUGCUGCUGGUAGCUUCA | Cleavage |
| osa-miR167b | OjGRF4 | 107 | 127 | UGAAGCUGCCAGCAUGAUCUA | GAGCUGCUGCUGGUAGCUUCA | Cleavage |
| osa-miR167c-5p | OjGRF4 | 107 | 127 | UGAAGCUGCCAGCAUGAUCUA | GAGCUGCUGCUGGUAGCUUCA | Cleavage |
| osa-miR167d-5p | OjGRF4 | 107 | 127 | UGAAGCUGCCAGCAUGAUCUG | GAGCUGCUGCUGGUAGCUUCA | Cleavage |
| osa-miR167e-5p | OjGRF4 | 107 | 127 | UGAAGCUGCCAGCAUGAUCUG | GAGCUGCUGCUGGUAGCUUCA | Cleavage |
| osa-miR167f | OjGRF4 | 107 | 127 | UGAAGCUGCCAGCAUGAUCUG | GAGCUGCUGCUGGUAGCUUCA | Cleavage |
| osa-miR167g | OjGRF4 | 107 | 127 | UGAAGCUGCCAGCAUGAUCUG | GAGCUGCUGCUGGUAGCUUCA | Cleavage |
| osa-miR167h-5p | OjGRF4 | 107 | 127 | UGAAGCUGCCAGCAUGAUCUG | GAGCUGCUGCUGGUAGCUUCA | Cleavage |
| osa-miR167i-5p | OjGRF4 | 107 | 127 | UGAAGCUGCCAGCAUGAUCUG | GAGCUGCUGCUGGUAGCUUCA | Cleavage |
| osa-miR167j | OjGRF4 | 107 | 127 | UGAAGCUGCCAGCAUGAUCUG | GAGCUGCUGCUGGUAGCUUCA | Cleavage |
| osa-miR5075 | OjGRF9 | 708 | 728 | UUCUCCGUCGCCGCCGUCCGC | CACGUCGGCGACGACGGAGAA | Translation |
| osa-miR171d-5p | OjGRF7 | 873 | 893 | UGUUGGCCCGGCUCACUCAGA | UCUGAGUGAGCUGGACCAGAA | Cleavage |
| osa-miR1874-3p | OjGRF11 | 417 | 440 | UAUGGAUGGAGGUGUAACCCGAUG | CAUCAGGAGAAGCCUCCAUCCAUG | Cleavage |
| osa-miR396f-5p | OjGRF5 | 337 | 358 | UCUCCACAGGCUUUCUUGAACU | CGUUCAAGAAAGCAUGUGGAAG | Cleavage |
| osa-miR396f-5p | OjGRF6 | 529 | 550 | UCUCCACAGGCUUUCUUGAACU | CGUUCAAGAAAGCCUAUGGAAG | Cleavage |
| osa-miR396f-5p | OjGRF11 | 583 | 604 | UCUCCACAGGCUUUCUUGAACU | CGUUCAAGAAAGCAUGUGGAAG | Cleavage |
| osa-miR396f-5p | OjGRF12 | 490 | 511 | UCUCCACAGGCUUUCUUGAACU | CGUUCAAGAAAGCAUGUGGAAG | Cleavage |
| osa-miR535-3p | OjGRF3 | 751 | 771 | GUGCUUUCUCCCGUUGUCACU | GAUGGCACCGAGAGGGAGCAC | Translation |
| osa-miR5819 | OjGRF9 | 578 | 598 | AGGACGAGGGGAACGGCGGCG | CGCCGCUCUUCCCGUCGUCCG | Cleavage |
| osa-miR5822 | OjGRF11 | 890 | 910 | UGUCUGCUCGAUGUCAGGUUG | ACAUCGGGCAUGGAGCAGGCA | Translation |
| osa-miR5832 | OjGRF3 | 1030 | 1050 | UUGGCGGAGCGGUUGCUGUCA | GCGCAGAAACCUCUCCGCCAC | Translation |
| osa-miR166l-5p | OjGRF12 | 39 | 59 | GGAUUGUUGUCUGGUUCAAGG | UCUUGGAUCAGGUGGCUAUCC | Cleavage |
| osa-miR167h-3p | OjGRF11 | 858 | 878 | AGGUCAUGCUGUAGUUUCAUC | ACUGAAGUUGCACCAUGAUCA | Cleavage |
| osa-miR171e-5p | OjGRF7 | 873 | 893 | UGUUGGCUCGGCUCACUCAGA | UCUGAGUGAGCUGGACCAGAA | Cleavage |
| osa-miR2927 | OjGRF9 | 701 | 723 | UGUCGUCGUCGAUGGAGCCCAUG | ACGACGCCACGUCGGCGACGACG | Cleavage |
| osa-miR396a-5p | OjGRF6 | 528 | 549 | UUCCACA-GCUUUCUUGAACUG | CCGUUCAAGAAAGCCUAUGGAA | Cleavage |
| osa-miR396b-5p | OjGRF6 | 528 | 549 | UUCCACA-GCUUUCUUGAACUG | CCGUUCAAGAAAGCCUAUGGAA | Cleavage |
| osa-miR396c-5p | OjGRF6 | 528 | 549 | UUCCACA-GCUUUCUUGAACUU | CCGUUCAAGAAAGCCUAUGGAA | Cleavage |
| osa-miR408-3p | OjGRF7 | 91 | 111 | CUGCACUGCCUCUUCCCUGGC | GUCGGGGAGGAGGCGCAGCAG | Cleavage |
| osa-miR5075 | OjGRF2 | 246 | 266 | UUCUCCGUCGCCGCCGUCCGC | GCCGCCGGCGGCGGCGGCGAG | Cleavage |
| osa-miR5075 | OjGRF3 | 1017 | 1037 | UUCUCCGUCGCCGCCGUCCGC | CCACGCGGCGGCGGCGCAGAA | Cleavage |
| osa-miR5075 | OjGRF8 | 18 | 38 | UUCUCCGUCGCCGCCGUCCGC | CUCGGCGGCGGCGCCGGGGAU | Cleavage |
| osa-miR5148a | OjGRF4 | 1057 | 1080 | UGAGGGGUAGAAAUGUCAUAUCAU | GUUCCAUCAACUUUCUAUUCCUCG | Cleavage |
| osa-miR5148b | OjGRF4 | 1057 | 1080 | UGAGGGGUAGAAAUGUCAUAUCAU | GUUCCAUCAACUUUCUAUUCCUCG | Cleavage |
| osa-miR5148c | OjGRF4 | 1057 | 1080 | UGAGGGGUAGAAAUGUCAUAUCAU | GUUCCAUCAACUUUCUAUUCCUCG | Cleavage |
| osa-miR5338 | OjGRF12 | 17 | 37 | UGAAGCUUCAGUUGGUUGUAU | CUACGACCAACGGCAGCUUCC | Translation |
| osa-miR5504 | OjGRF8 | 492 | 512 | AGUGACGGGAGGACUGCAAGG | CGAUGCGCCUCUCCCGUCACU | Cleavage |
| osa-miR5809 | OjGRF2 | 240 | 259 | UCGUCGCCGGCGACCACAGC | GAGGUGGCCGCCGGCGGCGG | Cleavage |
| osa-miR6246 | OjGRF11 | 738 | 760 | UUGGGGAUUUCCUGCCGGAGGAA | ACAAUCCAACAGGAAAUUUCUGG | Cleavage |
| osa-miR818f | OjGRF7 | 42 | 62 | GGGAGCAUGUAGGAUGGCCAU | CCGGCCCUCCUUCAUCUUCCC | Translation |
| osa-miR1440b | OjGRF2 | 676 | 696 | UUUAGGAGAGUGGUAUUUGAG | UUCCAAAACCACUCUCUUUAC | Cleavage |
| osa-miR166g-5p | OjGRF5 | 1237 | 1257 | AAUGGAGGCUGAUCCAAGAUC | ACCUUUGGAUCAGUAUCCAGU | Cleavage |
| osa-miR167c-3p | OjGRF4 | 127 | 149 | GGUCAUGCUGCGGCAGCCUCACU | ACCCAGGCUGCUGCUGCAUCAUA | Cleavage |
| osa-miR1857-5p | OjGRF7 | 383 | 403 | UGGUUUUUUUGGAGCAUGAGG | GGCGGUGCUCCAAGGAGGCCG | Cleavage |
| osa-miR1861a | OjGRF10 | 681 | 702 | UGAUCUUGAGGCAGAAACUGAG | GCCUGAUUCUACCUCAGGGUCG | Cleavage |
| osa-miR1861b | OjGRF10 | 681 | 702 | CGAUCUUGAGGCAGGAACUGAG | GCCUGAUUCUACCUCAGGGUCG | Cleavage |
| osa-miR1861f | OjGRF10 | 681 | 702 | CGAUCUUGAGGCAGGAACUGAG | GCCUGAUUCUACCUCAGGGUCG | Cleavage |
| osa-miR1861i | OjGRF10 | 681 | 702 | CGAUCUUGAGGCAGGAACUGAG | GCCUGAUUCUACCUCAGGGUCG | Cleavage |
| osa-miR1861l | OjGRF10 | 681 | 702 | CGAUCUUGAGGCAGGAACUGAG | GCCUGAUUCUACCUCAGGGUCG | Cleavage |
| osa-miR1861n | OjGRF2 | 840 | 861 | CGAUCUUGUGGCAGGAGCUGAG | UACUGCUUAUGGCACAAGAUCU | Translation |
| osa-miR1861o | OjGRF10 | 681 | 702 | UGAUCUUGAGGCAGAAACUGAG | GCCUGAUUCUACCUCAGGGUCG | Cleavage |
| osa-miR2055 | OjGRF10 | 35 | 55 | UUUCCUUGGGAAGGUGGUUUC | CCAGCAACCCUCCCGGGGGAG | Cleavage |
| osa-miR2093-5p | OjGRF4 | 815 | 834 | GUGCAUUAAUUGGAAGAACA | UGUUCUCUCAGUUGAAGCAA | Cleavage |
| osa-miR2096-5p | OjGRF10 | 44 | 64 | UGCCGAUUUCCCCCUCGGGCG | CUCCCGGGGGAGGAGGCGGCG | Translation |
| osa-miR2102-3p | OjGRF6 | 678 | 699 | CAUGGUGCCGGUUCCGGUGGCG | CCACACCACCACCGGCACCACG | Cleavage |
| osa-miR2275d | OjGRF10 | 581 | 602 | CUUGUUUUUCUCCAAUAUCUCA | GGCGGUGCUGGAGAAAUGCAAU | Cleavage |
| osa-miR2871a-3p | OjGRF12 | 110 | 130 | UAUUUUAGUUUCUAUGGUCAC | UGGAUUGUGGAAGCUCAGAUG | Cleavage |
| osa-miR2871b | OjGRF12 | 110 | 130 | UAUUUUAGUUUCUAUGGUCAC | UGGAUUGUGGAAGCUCAGAUG | Cleavage |
| osa-miR2872 | OjGRF12 | 106 | 126 | UGGGGUUCUACAAACCGAACU | GGGUUGGAUUGUGGAAGCUCA | Cleavage |
| osa-miR2919 | OjGRF4 | 145 | 163 | AAGGGGGGGGGGGGAAAGA | UCAUACCCUUCCCCCUUUC | Cleavage |
| osa-miR2925 | OjGRF7 | 508 | 526 | UGGCGGCCGCGGGCUUCGU | ACGGCGCCGGCCGCCGCCG | Translation |
| osa-miR395b | OjGRF5 | 801 | 821 | GUGAAGUGUUUGGGGGAACUC | AGUUUCCCUUCAACACUUUGU | Translation |
| osa-miR395d | OjGRF5 | 801 | 821 | GUGAAGUGUUUGGGGGAACUC | AGUUUCCCUUCAACACUUUGU | Translation |
| osa-miR395e | OjGRF5 | 801 | 821 | GUGAAGUGUUUGGGGGAACUC | AGUUUCCCUUCAACACUUUGU | Translation |
| osa-miR395g | OjGRF5 | 801 | 821 | GUGAAGUGUUUGGGGGAACUC | AGUUUCCCUUCAACACUUUGU | Translation |
| osa-miR395h | OjGRF5 | 801 | 821 | GUGAAGUGUUUGGGGGAACUC | AGUUUCCCUUCAACACUUUGU | Translation |
| osa-miR395i | OjGRF5 | 801 | 821 | GUGAAGUGUUUGGGGGAACUC | AGUUUCCCUUCAACACUUUGU | Translation |
| osa-miR395j | OjGRF5 | 801 | 821 | GUGAAGUGUUUGGGGGAACUC | AGUUUCCCUUCAACACUUUGU | Translation |
| osa-miR395k | OjGRF5 | 801 | 821 | GUGAAGUGUUUGGGGGAACUC | AGUUUCCCUUCAACACUUUGU | Translation |
| osa-miR395l | OjGRF5 | 801 | 821 | GUGAAGUGUUUGGGGGAACUC | AGUUUCCCUUCAACACUUUGU | Translation |
| osa-miR395m | OjGRF5 | 801 | 821 | GUGAAGUGUUUGGGGGAACUC | AGUUUCCCUUCAACACUUUGU | Translation |
| osa-miR395n | OjGRF5 | 801 | 821 | GUGAAGUGUUUGGGGGAACUC | AGUUUCCCUUCAACACUUUGU | Translation |
| osa-miR395p | OjGRF5 | 801 | 821 | GUGAAGUGUUUGGGGGAACUC | AGUUUCCCUUCAACACUUUGU | Translation |
| osa-miR395q | OjGRF5 | 801 | 821 | GUGAAGUGUUUGGGGGAACUC | AGUUUCCCUUCAACACUUUGU | Translation |
| osa-miR395r | OjGRF5 | 801 | 821 | GUGAAGUGUUUGGGGGAACUC | AGUUUCCCUUCAACACUUUGU | Translation |
| osa-miR395s | OjGRF5 | 801 | 821 | GUGAAGUGUUUGGGGGAACUC | AGUUUCCCUUCAACACUUUGU | Translation |
| osa-miR395y | OjGRF5 | 801 | 821 | GUGAAGUGUUUGGGGGAACUC | AGUUUCCCUUCAACACUUUGU | Translation |
| osa-miR399h | OjGRF3 | 224 | 244 | UGCCAAAGGAGACUUGCCCAG | UGGGGUGGGGUUGCUUUGGCA | Cleavage |
| osa-miR399k | OjGRF3 | 224 | 244 | UGCCAAAGGAAAUUUGCCCCG | UGGGGUGGGGUUGCUUUGGCA | Cleavage |
| osa-miR426 | OjGRF11 | 983 | 1003 | UUUUGGAAGUUUGUCCUUACG | CUUUAGGUGAAGUUUUCAAAA | Cleavage |
| osa-miR5073 | OjGRF12 | 959 | 981 | GUUUGGUGAAUCGGAAACUAUUU | AUGUAGUAGGCGGUUCAUCGAAU | Cleavage |
| osa-miR5075 | OjGRF9 | 222 | 242 | UUCUCCGUCGCCGCCGUCCGC | UGGCGCGGCGGCGGCGCAGUA | Cleavage |
| osa-miR5075 | OjGRF3 | 22 | 42 | UUCUCCGUCGCCGCCGUCCGC | CCGAGCGGCGGCGCCGGCGGA | Cleavage |
| osa-miR5075 | OjGRF1 | 174 | 194 | UUCUCCGUCGCCGCCGUCCGC | GUCGGCGGCGGCGGCGCACAA | Cleavage |
| osa-miR5075 | OjGRF10 | 55 | 75 | UUCUCCGUCGCCGCCGUCCGC | GGAGGCGGCGGAGGUGGAGGG | Translation |
| osa-miR530-3p | OjGRF11 | 379 | 399 | AGGUGCAGAGGCAGAUGCAAC | GCUGCAAAUGUUUCUGUCCCU | Cleavage |
| osa-miR531a | OjGRF1 | 137 | 159 | CUCGCCGGGGCUGCGUGCCGCCAU | UCGGCGGCGA-CAGCCGCGGCGAG | Cleavage |
| osa-miR531b | OjGRF1 | 141 | 159 | CUCGCCGGGGCUGCGUGCCG | CGGCGA-CAGCCGCGGCGAG | Cleavage |
| osa-miR531c | OjGRF1 | 137 | 159 | CUCGCCGGGGCUGCGUGCCGCCAU | UCGGCGGCGA-CAGCCGCGGCGAG | Cleavage |
| osa-miR5338 | OjGRF4 | 368 | 388 | UGAAGCUUCAGUUGGUUGUAU | UUUCAAUCAGCAAAAGCUUCA | Translation |
| osa-miR5338 | OjGRF4 | 1008 | 1028 | UGAAGCUUCAGUUGGUUGUAU | GGACGAGAAAAUGGAGUUUCA | Translation |
| osa-miR5526 | OjGRF10 | 676 | 696 | AAAGGUAGAGUCAGGUAUGAG | GACGAGCCUGAUUCUACCUCA | Cleavage |
| osa-miR5542 | OjGRF11 | 718 | 737 | UUUGAGAAGGUAUCAUGAGAU | GUCUC-UGAUCCUUUCUCGAG | Translation |
| osa-miR6248 | OjGRF5 | 641 | 661 | UAUUUGAGGAUGGAGGUAGUA | GCCUGUUUCCGUUCUCAAAAC | Cleavage |
| osa-miR6249a | OjGRF12 | 1042 | 1062 | CGUGAAGAGCUCGCCGGCGGC | CCUCUGGGUGAGUUCUUCACC | Cleavage |
| osa-miR6249b | OjGRF12 | 1042 | 1062 | CGUGAAGAGCUCGCCGGCGGC | CCUCUGGGUGAGUUCUUCACC | Cleavage |
|  |  |  |  |  |  |  |
|  |  |  |  |  |  |  |

**Supplementary Table 8** List of oligonucleotide primers used for qRT-PCR assays

| Primer name | Forward sequence (5'-3') | Reverse sequence (5'-3') |
| --- | --- | --- |
| RT-qPCR-OjGRF1 | TTTGGGCGGAAGGCGGAGGA | TTGCCACGGTGCATGTGCTT |
| RT-qPCR-OjGRF2 | CAAGAAAGCCTGTGGAAATGCCG | GCGTGGAAGGAGTCGAGGTGGA |
| RT-qPCR-OjGRF3 | CGCAACCGTTCAAGAAAGCC | TAGCAATCGCCGGGTACAGC |
| RT-qPCR-OjGRF4 | TTCAAGAAAGCCTGTGGAAACG | CCGGCAATAGCAGGGTAAAGA |
| RT-qPCR-OjGRF5 | GCCTCCTACCAGGTGCCTACTC | GGTTCTCGTGCTTCACATTTCC |
| RT-qPCR-OjGRF6 | TCATCAGCCTCAACATCTCC | CATCCAGTTTGCTTCTCCCT |
| RT-qPCR-OjGRF7 | GCTGGGCTATTCTGGGAGTG | ATGCTTTCTTGAACGGTGGC |
| RT-qPCR-OjGRF8 | GATCATTCCCTCCTGGCTGTG | ATGGTGAGTGTCGCCTTTCG |
| RT-qPCR-OjGRF9 | GGAGAAGTTCAATACAGCAGGAG | CTGACTGAAGGCATCCACGA |
| RT-qPCR-OjGRF10 | CCCGAATGCCGGAGTGGTGA | GCTCCTGCTGCTGCATGAACGT |
| RT-qPCR-OjGRF11 | TGTGCCTGTGCCTACTCATCTC | GGTTCTGGTTCTGGGTTCTTTC |
| RT-qPCR-OjGRF12 | TCTTGGGCTGGGTCTCGGTCTG | GCGGCGAAGTAGCGGTAGATGAG |
| RT-qPCR-OsIAA1 | ACCAAGAGCCGCTCAATGAG | ATCACACGTGGGCGAACATC |
| RT-qPCR-OsIAA8 | GACGGCAACAACCCTTCT | GGTGATGGATGCTCTGAAC |
| RT-qPCR-OsIAA11 | GCGCTGGTGAAGGTGAGCAT | ACGTACTCCAGGTCATCTCT |
| RT-qPCR-OsIAA20 | CATCCTCGGCTCATACGC | ATCGTGCCCATCCTCTTG |
| RT-qPCR-OsIAA23 | AAGCTGGTCAAGGTGGCC | TCCAGTCGCCGTCCTTGT |
| RT-qPCR-OsIAA31 | GCAGCAGAAGGAGGATGT | CGGAGAAGCAGAGGAACAT |
| RT-qPCR-OsAUX1 | GCCTGCGCGAGTAACATCTA | CAGCACCAGCTTGGTTGGAC |
| RT-qPCR-OsAUX3 | CTGCTGCTGCAGGTGTTCTACG | GTATATGTTACTTGCACAGGCGAT |
| RT-qPCR-OsAUX4 | GGCCTTCAACTGCACGTTTT | GATGTACCAGGCGGTGTAGG |

**Supplementary Table 9** List of oligonucleotide primers used for subcellular localization analysis

| Primer name | Forward sequence (5'-3') | Reverse sequence (5'-3') |
| --- | --- | --- |
| subcellular-OjGRF1 | CCTCTCCCCTTGCTCCGTATGGATGAGGAGAAGGAAGCC | CTCGCCCTTGCTCACCATGGATCCATCCGGTGGCGCGCGCGGTGCCGTGG |
| subcellular-OjGRF2 | CCTCTCCCCTTGCTCCGTATGCCTCCCTGTCTCCGGCGG | CTCGCCCTTGCTCACCATGGATCCGTCACCATTAGTTGATCGAGA |
| subcellular-OjGRF3 | CCTCTCCCCTTGCTCCGTATGATGATGATGAGCGGTCG | CTCGCCCTTGCTCACCATGGATCCATCATCATTGTGGTAGCGGGA |
| subcellular-OjGRF5 | CCTCTCCCCTTGCTCCGTATGCAGGGTGCAATGGCCAGGGTG | CTCGCCCTTGCTCACCATGGATCCCACCAGGCGGATGCTCGGATGATT |
| subcellular-OjGRF12 | CCTCTCCCCTTGCTCCGTATGGCAATGGCGACCCCTACG | CTCGCCCTTGCTCACCATGGATCCCAGTGTTTGGACAGTCTGGGCATTCACC |

**Supplementary Table 10** List of oligonucleotide primers used for transcriptional activity assays

| Primer name | Forward sequence (5'-3') | Reverse sequence (5'-3') |
| --- | --- | --- |
| GAL4DB-OjGRF1 | CCCCCGGGCTGCAGGAATTCATGGATGAGGAGAAGGAAGCC | ATCGATAAGCTTGATATCGAATTCGGTGGCGCGCGCGGTGCCGTGG |
| GAL4DB-OjGRF2 | CCCCCGGGCTGCAGGAATTCATGCCTCCCTGTCTCCGGCGG | ATCGATAAGCTTGATATCGAATTCGTCACCATTAGTTGATCGAGA |
| GAL4DB-OjGRF3 | CCCCCGGGCTGCAGGAATTCATGATGATGATGAGCGGTCG | ATCGATAAGCTTGATATCGAATTCATCATCATTGTGGTAGCGGGA |
| GAL4DB-OjGRF4 | CCCCCGGGCTGCAGGAATTCATGTTTGCTGACTTCTCTGCT | ATCGATAAGCTTGATATCGAATTCCATTGCATGATCCTCGAGGTT |
| GAL4DB-OjGRF5 | CCCCCGGGCTGCAGGAATTCATGCAGGGTGCAATGGCCAGGGTG | ATCGATAAGCTTGATATCGAATTCCACCAGGCGGATGCTCGGATGATT |
| GAL4DB-OjGRF6 | CCCCCGGGCTGCAGGAATTCATGTTGGCCGAGGGAAGGCAA | ATCGATAAGCTTGATATCGAATTCAGTAGCGCGCGTGGTGCCGGTGG |
| GAL4DB-OjGRF7 | CCCCCGGGCTGCAGGAATTCATGGCGATGCCCTTTGCCTCCC | ATCGATAAGCTTGATATCGAATTCCCGGGAGTATATACCGTTGTG |
| GAL4DB-OjGRF8 | CCCCCGGGCTGCAGGAATTCATGCTGAGCTCGTCGCCCTCG | ATCGATAAGCTTGATATCGAATTCAGGTAGACGCGGACCGATAGG |
| GAL4DB-OjGRF9 | CCCCCGGGCTGCAGGAATTCATGATGGCCGGCGGCGGATCG | ATCGATAAGCTTGATATCGAATTCCTCGCTCGTCCGCAGATGGTC |
| GAL4DB-OjGRF10 | CCCCCGGGCTGCAGGAATTCATGGCGGCGGAGGGGGAGGCC | ATCGATAAGCTTGATATCGAATTCTGTAGACTCCACAGCAGCTGGT |
| GAL4DB-OjGRF11 | CCCCCGGGCTGCAGGAATTCATGCTGAGCTCTTGTGGTGGC | ATCGATAAGCTTGATATCGAATTCGAGAAGTGTTGGGACAATATG |
| GAL4DB-OjGRF12 | CCCCCGGGCTGCAGGAATTCATGGCAATGGCGACCCCTACG | ATCGATAAGCTTGATATCGAATTCCAGTGTTTGGACAGTCTGGGCATTCACC |

**Supplementary Table 11** List of oligonucleotide primers used for yeast assays

| Primer name | Forward sequence (5'-3') | Reverse sequence (5'-3') |
| --- | --- | --- |
| AD-OjGRF1 | GCCATGGAGGCCAGTGAATTCATGGATGAGGAGAAGGAAGCC | CAGCTCGAGCTCGATGGATCCCGGTGGCGCGCGCGGTGCCGTGG |
| AD-OjGRF2 | GCCATGGAGGCCAGTGAATTCATGCCTCCCTGTCTCCGGCGG | CAGCTCGAGCTCGATGGATCCCGTCACCATTAGTTGATCGAGA |
| AD-OjGRF3 | GCCATGGAGGCCAGTGAATTCATGATGATGATGAGCGGTCG | CAGCTCGAGCTCGATGGATCCCATCATCATTGTGGTAGCGGGA |
| AD-OjGRF4 | GCCATGGAGGCCAGTGAATTCATGTTTGCTGACTTCTCTGCT | CAGCTCGAGCTCGATGGATCCCCATTGCATGATCCTCGAGGTT |
| AD-OjGRF5 | GCCATGGAGGCCAGTGAATTCATGCAGGGTGCAATGGCCAGGGTG | CAGCTCGAGCTCGATGGATCCCCACCAGGCGGATGCTCGGATGATT |
| AD-OjGRF6 | GCCATGGAGGCCAGTGAATTCATGTTGGCCGAGGGAAGGCAA | CAGCTCGAGCTCGATGGATCCCAGTAGCGCGCGTGGTGCCGGTGG |
| AD-OjGRF7 | GCCATGGAGGCCAGTGAATTCATGGCGATGCCCTTTGCCTCCC | CAGCTCGAGCTCGATGGATCCCCCGGGAGTATATACCGTTGTG |
| AD-OjGRF8 | GCCATGGAGGCCAGTGAATTCATGCTGAGCTCGTCGCCCTCG | CAGCTCGAGCTCGATGGATCCCAGGTAGACGCGGACCGATAGG |
| AD-OjGRF9 | GCCATGGAGGCCAGTGAATTCATGATGGCCGGCGGCGGATCG | CAGCTCGAGCTCGATGGATCCCCTCGCTCGTCCGCAGATGGTC |
| AD-OjGRF10 | GCCATGGAGGCCAGTGAATTCATGGCGGCGGAGGGGGAGGCC | CAGCTCGAGCTCGATGGATCCCTGTAGACTCCACAGCAGCTGGT |
| AD-OjGRF11 | GCCATGGAGGCCAGTGAATTCATGCTGAGCTCTTGTGGTGGC | CAGCTCGAGCTCGATGGATCCCGAGAAGTGTTGGGACAATATG |
| AD-OjGRF12 | GCCATGGAGGCCAGTGAATTCATGGCAATGGCGACCCCTACG | CAGCTCGAGCTCGATGGATCCCCAGTGTTTGGACAGTCTGGGCATTCACC |
| BD-OjGRF1 | ATGGCCATGGAGGCCGAATTCATGGATGAGGAGAAGGAAGCC | GGTTATGCTAGTTATGCGGCCGCGGTGGCGCGCGCGGTGCCGTGG |
| BD-OjGRF2 | ATGGCCATGGAGGCCGAATTCATGCCTCCCTGTCTCCGGCGG | GGTTATGCTAGTTATGCGGCCGCGTCACCATTAGTTGATCGAGA |
| BD-OjGRF3 | ATGGCCATGGAGGCCGAATTCATGATGATGATGAGCGGTCG | GGTTATGCTAGTTATGCGGCCGCATCATCATTGTGGTAGCGGGA |
| BD-OjGRF4 | ATGGCCATGGAGGCCGAATTCATGTTTGCTGACTTCTCTGCT | GGTTATGCTAGTTATGCGGCCGCCATTGCATGATCCTCGAGGTT |
| BD-OjGRF5 | ATGGCCATGGAGGCCGAATTCATGCAGGGTGCAATGGCCAGGGTG | GGTTATGCTAGTTATGCGGCCGCCACCAGGCGGATGCTCGGATGATT |
| BD-OjGRF6 | ATGGCCATGGAGGCCGAATTCATGTTGGCCGAGGGAAGGCAA | GGTTATGCTAGTTATGCGGCCGCAGTAGCGCGCGTGGTGCCGGTGG |
| BD-OjGRF7 | ATGGCCATGGAGGCCGAATTCATGGCGATGCCCTTTGCCTCCC | GGTTATGCTAGTTATGCGGCCGCCCGGGAGTATATACCGTTGTG |
| BD-OjGRF8 | ATGGCCATGGAGGCCGAATTCATGCTGAGCTCGTCGCCCTCG | GGTTATGCTAGTTATGCGGCCGCAGGTAGACGCGGACCGATAGG |
| BD-OjGRF9 | ATGGCCATGGAGGCCGAATTCATGATGGCCGGCGGCGGATCG | GGTTATGCTAGTTATGCGGCCGCCTCGCTCGTCCGCAGATGGTC |
| BD-OjGRF10 | ATGGCCATGGAGGCCGAATTCATGGCGGCGGAGGGGGAGGCC | GGTTATGCTAGTTATGCGGCCGCTGTAGACTCCACAGCAGCTGGT |
| BD-OjGRF11 | ATGGCCATGGAGGCCGAATTCATGCTGAGCTCTTGTGGTGGC | GGTTATGCTAGTTATGCGGCCGCGAGAAGTGTTGGGACAATATG |
| BD-OjGRF12 | ATGGCCATGGAGGCCGAATTCATGGCAATGGCGACCCCTACG | GGTTATGCTAGTTATGCGGCCGCCAGTGTTTGGACAGTCTGGGCATTCACC |
